# The exposure of Czech firefighters to perfluoroalkyl substances and polycyclic aromatic hydrocarbons: CELSPAC – FIREexpo case-control human biomonitoring study

**DOI:** 10.1016/j.scitotenv.2023.163298

**Published:** 2023-07-10

**Authors:** Katarína Řiháčková, Aleš Pindur, Klára Komprdová, Nina Pálešová, Jiří Kohoutek, Petr Šenk, Jana Navrátilová, Lenka Andrýsková, Ludmila Šebejová, Richard Hůlek, Mazen Ismael, Pavel Čupr

**Affiliations:** a RECETOX, Faculty of Science, Masaryk University, Kamenice 753/5, 625 00 Brno, Czech Republic; b Faculty of Sports Studies, Masaryk University, Kamenice 753/5, 625 00 Brno, Czech Republic; c Training Center of Fire Rescue Service, General Directorate of Fire Rescue Service of the Czech Republic, Ministry of the Interior of the Czech Republic, Trnkova 85, 628 00 Brno, Czech Republic

**Keywords:** Firefighters, Human biomonitoring, Perfluoroalkyl substances, Polycyclic aromatic hydrocarbons, Cohort profile, HBM value

## Abstract

The CELSPAC – FIREexpo biomonitoring study investigates the long-term effects of chemical exposure on firefighters' wellness and fitness. It aims to provide science-based measures to minimize the health risks of the firefighting occupation. Here, we present the study design, cohort profile, and first results with respect to internal per- and polyfluoroalkyl substances (PFAS) and polycyclic aromatic hydrocarbons (PAH) levels in study participants. Participants (n = 166) were divided into three subcohorts: i) newly recruited firefighters, ii) professional firefighters with several years' experience, and iii) the control group. Participants underwent physical performance tests, provided information on their lifestyle and diet, and urine and blood samples 1–4 times within an 11-week period. 12 serum PFAS and 10 urinary hydroxylated PAH (OH-PAH) levels were determined using HPLC-MS/MS and compared between subcohorts and samplings. The association of internal exposure with reported lifestyles and occupational factors was investigated using Spearman's correlation, principal component analysis, and multivariate regression analysis. ΣPFAS levels in firefighters were significantly higher than in the control group and were mostly associated with the length of firefighting career, age, blood donation, and population size. 10.9 % and 7.6 % of measurements exceeded the HBM-I or HBM-II value for PFOS and PFOA, respectively. Urinary ΣPAH levels increased significantly after training with burning wooden pallets, but none of them exceeded the no observed genotoxic effect level. Firefighters’ occupational exposure, its sources, and pathways, need to be systematically monitored and investigated on a long-term and individual basis. The CELSPAC – FIREexpo study helps to clarify the degree of occupational exposure to the given compounds and the subsequent risks to firefighters.

## Highlights

• Firefighters had significantly higher serum PFAS levels than the control group.
• One training with firefighting foam did not significantly increase PFAS levels.
• 10.9 % and 7.6 % of samples exceeded the HBM-I values for PFOS and PFOA.
• Career length or age, and blood donation affected firefighters’ serum PFAS levels.
• Training with burning wooden pallets significantly increased urine OH-PAH levels.

## Graphical abstract

**Figure:**
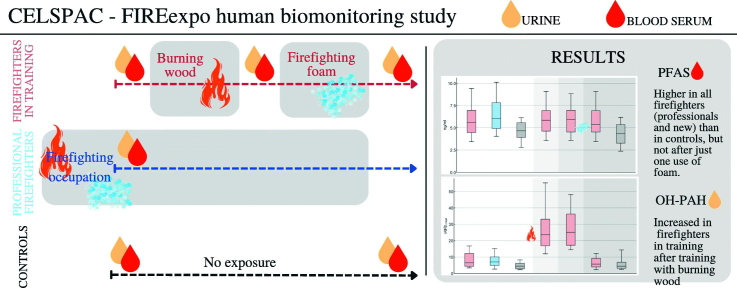

## 1 Introduction

Firefighters are exposed to many factors that can reduce their fitness and wellness, and adversely affect their health (Schnell et al., 2020; Soteriades et al., 2011; Gianniou et al., 2016; Navarro et al., 2019). Hazardous chemicals and their mixtures are among the most pronounced ones. Some of them emerge undesirably during an incident, e.g. polycyclic aromatic hydrocarbons (PAH), products of incomplete combustion of organic matter. Others are intentionally used. These are for example the per- and polyfluoroalkyl substances (PFAS) contained in some firefighting foams or personal protective equipment. Many studies have already presented evidence that the occupation of firefighting and firefighting training can lead to increased internal exposure to PAH, PFAS, and other compounds (Banks et al., 2021; Fent et al., 2019; Laitinen et al., 2010; Nilsson et al., 2022a; Trowbridge et al., 2020; Dobraca et al., 2015a) which, in turn, can lead to adverse health effects including cancer, reduced fertility, and neurotoxicity (IARC, 2010; Kamangar et al., 2005; Moorthy et al., 2015; Banks et al., 2014; Cao and Ng, 2021; Steenland and Winquist, 2021; Petersen et al., 2020).

Approximately 10,100 out of 14,200 professional firefighters in the Czech Republic can potentially be involved in first responses to incidents (data to 2020) (Nedělníková, 2021), and therefore exposed to the risk of death, injury, or exposure to chemicals and other agents. This number represents almost 0.1 % of the Czech population. Moreover, there are >64,200 registered voluntary firefighters in the Czech Republic (data to 2020), all of them potentially involved in first response (Nedělníková, 2021). While injuries and deaths of firefighters in response to incidents are well recorded, there is a lack of data and understanding on firefighters' chronic exposure to physical, mental, and chemical stressors and their links with health.

Human biomonitoring is an effective tool to determine aggregated exposure to chemical mixtures (Ganzleben et al., 2017; EEA-JRC, 2013). It enables the estimation of health risks resulting from chemical exposure, and their evolution in time. It facilitates evaluation of the efficiency of existing policies and creates new effective policies to manage chemicals and protect human health (Ganzleben et al., 2017; EEA-JRC, 2013). Although much attention has been paid worldwide to evaluating occupational exposure and resulting risks to firefighters, no complex human biomonitoring study has been carried out in the Czech Republic or anywhere in Central or Eastern Europe to date. Extrapolating knowledge from studies carried out in other regions might incorporate uncertainty because of regional differences in occupation and lifestyle factors affecting the exposure, as well as legislation and policies concerning the studied compounds.

Therefore, the CELSPAC – FIREexpo case-control human biomonitoring study was established in 2018 (RECETOX, 2022) with the following aims: i) to determine the effect of the firefighting profession and training on internal levels of PFAS, PAH, flame retardants, and other compounds; ii) to determine the effect of the firefighting profession and training on health related markers; iii) to link the markers of internal exposure, effect, and health, and iv) to propose science-based measures and recommendations to prevent or mitigate the risks of the firefighting profession.

In this research article, we present the CELSPAC – FIREexpo study design and cohort profile and focus on quantification of the firefighters' internal exposure to PFAS and PAH. We also investigate the factors involved in the firefighters' occupation, training, and lifestyle which might be associated with these PFAS and PAH levels.

## 2 Material and methods

### 2.1 Study population

The study was approved by the ELSPAC Ethics Committee, and launched in January 2019. Participants were approached and recruited in person and by means of leaflets distributed in the Training Center of the Fire Rescue Service, the Fire Rescue Service of the Czech Republic, and the Faculty of Sports Studies of Masaryk University. All participants expressed and signed their informed consent before their participation in the study. A smoking habit, chronic or acute infectious disease, mental illness, acute musculoskeletal injury, and age <18 or >35 years were all exclusion criteria. All participants were non-smoking White males from 18 to 35 years old. This population subgroup is the most accurate representation of current professional firefighters actively participating in the response to incidents in the Czech Republic.

The participants were divided into three groups (subcohorts): i) firefighters who had been recently employed in the Fire Rescue Service of the Czech Republic and who had not yet participated in the training to qualify for active participation in the response to incidents (new, n = 59); ii) professional firefighters who had been actively participating in the response to incidents (have participated in at least 2 times in extinguishing fire) (prof, n = 52); and iii) a control group comprising general population (non-firefighters) (ctrl, n = 55). One participant from group ii) and one from group iii) suspended their participation in the study due to health issues. The minimum number of 50 participants per group was calculated on the basis of literature and pilot study data to obtain sufficient statistical power for determining the impact of occupational and training activities on biomarkers and for making significant comparisons among groups. Participants were from within the whole Czech Republic. The new trainee's training took place in the training center in Brno, Czech Republic. Professional firefighters were coming from several fire rescue stations, both urban and rural, across the whole Czech Republic. All samples, questionnaires, and data were pseudonymized to protect the identity of the participants. Table 1 shows the baseline cohort characteristics.

**Table 1:** Baseline characteristics for each studied group (new = newly recruited firefighters in training, prof = professional firefighters who participate in the response to incidents, ctrl = control group).

		new	prof	ctrl
Number of participants recruited to the study		59	52	55
Age (years)	Median	24.5	28.0	26.0
	10th–90th perc.	21–31	23–33	20–32
	Min.–Max.	19–34	20–35	18–35
BMI (kg/m^2^)	Median	26.3	26.2	24.6
	10th–90th perc.	22.6–30.3	22.9–29	21.6–28.6
	Min.–Max.	20.7–33.4	21.1–32.2	18.4–30.9
Infectious or chronic disease (%)	Yes	0.0	0.0	5.5
	No	100.0	100.0	94.6
Health (subjective assessment, %)	Always healthy and well	50.9	67.3	56.4
	Mostly healthy and well	49.2	32.7	41.8
	Often do not feel well	0.0	0.0	1.8
Job (%)	Firefighter	100.0	100.0	0.0
	Student	0.0	0.0	45.5
	IT	0.0	0.0	14.6
	Office	0.0	0.0	12.7
	Other	0.0	0.0	27.2
Length of a firefighting career (years)	Median	0.5	3.3	0.0
	10th–90th perc.	0.25–1	1–10	0.0
	Min.–Max.	0–5	0.5–14	0.0
Smoking (%)	Yes	0.0	0.0	0.0
	No	100.0	100.0	100.0
How many of the non-smokers are former smokers (%)	Yes	11.9	19.2	7.3
	No	88.1	80.8	92.7
If a former smoker, years since quitting (years)	Median	4.0	0.5	3.5
	10th–90th perc.	0.5–10	0.2–5	0.6–8
	Min.–Max.	0.5–10	0.1-6	0.6–8
Contact with a large fire in the last 6 months (%)	Two or more times	22.0	59.6	5.5
	One time	32.2	21.2	0.0
	Never	45.8	19.2	90.9
Contact with firefighting foams in the last year (%)	Two or more times	0.0	34.6	0.0
	One time	25.4	40.4	1.8
	Never	69.5	25.0	98.2

### 2.2 Study design

**Fig. 1:**
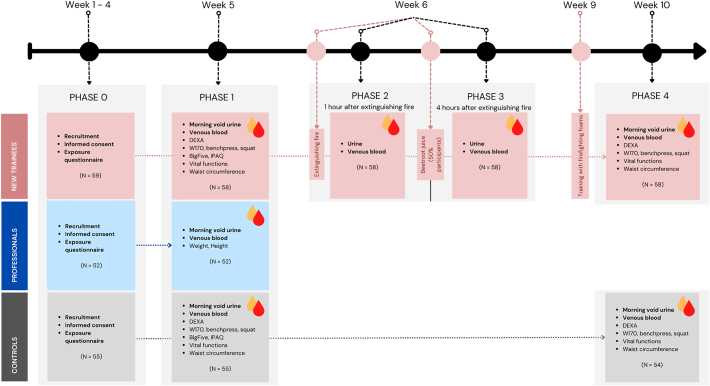
CELSPAC - FIREexpo study design. In total, 166 participants were recruited and divided into three subcohorts: New firefighters in training (new), professional firefighters (prof), and the control group (ctrl). The sampling protocol was structured into four phases during the 11 weeks, corresponding with the training period of the new firefighters (the control group and the professional firefighters did not undergo this training during the study).

The sampling campaign took place from January 2019 to June 2020 in several batches of 6–10 participants. New trainees must complete a 15-week training program to become eligible for participation in response to incidents. Therefore, the sampling protocol was structured into 4 phases covering the first eleven weeks of training that involves activities that were expected to affect the studied markers (Fig. 1). Only new trainees underwent the training and four phases of testing/samplings within the training period. Professionals only underwent the testing in the first phase. The control group was tested in the first, and last phases of the training.

The recruitment phase (Phase 0) took place in the 1st – 4th week of training. The first tests took place in Phase 1 (5th week of the training). Phases 2 and 3 took place on one day during the 6th week of the training. Phase 4, the final phase, took place in the 10th week of the training.

Participants were recruited, signed the informed consent, and filled out the exposure questionnaires in phase 0 of the study. In phase 1, all participants provided morning void urine samples and venous blood samples for the further analysis of biomarkers. Additionally, new trainees and the control group underwent an analysis of body composition using Dual X-ray absorptiometry (DEXA), tests of physical performance (Physical Work Capacity 170 test (PWC170), a bench press test, muscular strength one repetition maximum 1RM squat), and the measurement of life functions and waist circumference, and also completed the International Physical Activity Questionnaire (IPAQ) and the NEO-PI-R five-factor personality inventory (Big Five personality traits). The anthropometric parameters, DEXA, physical tests, IPAC, and Big Five inventory were carried out in the Masaryk university laboratories and at the Training Center of the Fire Rescue Service of the Czech Republic under the supervision of medical personnel and a psychologist. The professional firefighters reported their height and weight to calculate their BMI, and the BMI of controls and new trainees was calculated using the values of height and weight measured by DEXA. BMI was calculated by dividing the weight by the square of height.

Phases 2 and 3 corresponded with samplings 1 h and 4 h after the end of training with burning wooden pallets in a closed container and only applied to new trainees. In this training phase, no firefighting foam was used. The new trainees provided urine and venous blood samples in both phases. In addition, 33 out of 58 of the new trainees drank commercially available concentrated beetroot juice after the training between phases 2 and 3 of the study to later study the effect of vitamines and antioxidant supplements on the health markers.

Lastly, in phase 4, all new trainees and controls provided venous blood samples and morning void urine samples and underwent tests of physical performance (W170, bench press, and squat) and an analysis of body composition (DEXA). This phase did not apply to professional firefighters. For the new trainees, phase 4 was conducted 2 weeks after training in extinguishing flammable liquids using firefighting foams containing PFAS. A detailed description of the training activities and the equipment of trainees is provided in the Supplementary material.

### 2.3 Exposure questionnaires and population size

Upon inclusion in the study, all participants filled out exposure questionnaires about lifestyle and dietary factors possibly contributing to PAH exposure (former smoking habit, years since quitting smoking, proximity or exposure to fire, type of heating at home, and kind of heating fuel), PFAS exposure (frequency of wearing clothing with GoreTex or eVENT membranes, work in the ski service sector, skiing activities, self-service preparation of skis for winter, sources of drinking water at home and work (water well, mains water, bottled water), use of a filtration device for drinking water, use of dental floss, and blood donation), or exposure to both PAH and PFAS (length of firefighting career where relevant, diet and frequency of consumption of relevant food types and food supplements). The questionnaires also solicited information on the presence of acute or chronic infectious disease (at the time of filling the questionnaire), the participant's subjective health assessment, and employment. In addition, participants provided any other contextual information about their lifestyle and diet that might be relevant to the study.

Publicly available data from the population census current to the date 1. 1. 2019, collected by the Czech Statistical Office (CZSO) (Czech Statistical Office, 2019), were used as a source of population size for the places of residence of all participants. The number of inhabitants was matched using the address and postal code provided by participants during their registration for the study. The number of inhabitants was rounded to the nearest hundred.

### 2.4 Blood and urine collection

Blood samples were collected by medical personnel in an operational ambulance. Urine samples were collected at the workplace by own urine collection following the instruction of medical personnel. In phases 1 and 4, morning void midstream urine was sampled, along with venous blood on an empty stomach. In phases 2 and 3, the sampling of morning void urine and venous blood on an empty stomach was not possible due to the training schedule.

Venous blood for serum isolation was sampled in a 7.5 ml S-Monovette® tube containing the *Z*-gel clotting activator. Approximately 40 ml of midstream urine was collected by each participant using a 50 ml centrifuge tube. The venous blood and urine samples were immediately transported to laboratories in a cooling box (at 8 °C).

After a clot had emerged, the tube with venous blood was centrifuged for 10 min at 2500 ×*g* and 20 °C. 0.5 ml aliquots of serum were then divided into 1.2 ml cryotubes, frozen gradually, and stored in a biobank facility at −80 °C until further analyses of the biomarkers of exposure and effects and biochemical analysis.

The urine samples in 50 ml centrifuge tubes were divided into 1 ml aliquots in 1.2 ml cryotubes, frozen gradually, and stored in a biobank facility at −80 °C until further analyses.

### 2.5 Chemical analysis

The chemical analysis was undertaken two times when a sufficient number of samples was collected (i.e. samples were analyzed in two batches).

OH-PAH, the metabolites of PAH, namely 1-hydroxynaphthalene (1-OH-Naph), 2-hydroxynaphthalene (2-OH-Naph), 2-hydroxyfluorene (2-OH-Fluo), 3-hydroxyfluorene (3-OH-Fluo), 2/3-hydroxyphenanthrene (2/3-OH-Phen), 9-hydroxyphenanthrene (9-OH-Phen), 1-hydroxyphenanthrene (1-OH-Phen), 4-hydroxyphenanthrene (4-OH-Phen), 1-hydroxypyrene (1-OH-Pyr), and 3-hydroxybenzo[*a*]pyrene (3-OH-BaP), were analyzed using protocol based on CDC method 6705.02 (CDC's National Center for Environmental Health (NCEH), n.d.). Laboratory and method performance was successfully verified by participation in third-party proficiency testing (ICI-EQUAS, OSEQAS).

PFAS, namely perfluoropentanoic acid (PFPA), perfluorohexanoic acid (PFHxA), perfluoroheptanoic acid (PFHpA), perfluorooctanoic acid (PFOA), perfluorononanoic acid (PFNA), perfluorodecanoic acid (PFDA), perfluoroundecanoic acid (PFUnDA), perfluorododecanoic acid (PFDoDA), perfluorobutanoic acid (PFBA), perfluorohexane sulfonic acid (PFHxS), perfluoroheptane sulfonic acid (PFHpS), and perlfuorooctane sulfonate (PFOS), were analyzed using using protocol based on the CDC method 6304.04.(CDC's National Center for Environmental Health (NCEH), 2013) Laboratory and method performance was successfully verified in third-party proficiency testing (EQUAS, AMAP).

Urine creatinine levels and specific gravity were determined for the urine OH-PAH levels adjustment. Creatinine levels in urine were determined by LC-MS/MS using a modified procedure described by Dereziński et al. (Dereziński et al., 2016). Urine specific gravity (SG) was determined using a refractometer.

A detailed description of the analytical procedures, including QA/QC, is in the Supplementary material, section Chemical analysis.

### 2.6 Data analysis

Before statistical analysis, values below the limit of detection (<LOD) were substituted by the value of LOD/√2. Both creatinine and SG adjustment (Table SI02) was applied to urine OH-PAH concentrations. The levels of creatinine and SG are in the Supplementary material, Table SI03. Creatinine and SG were strongly correlated (r > 0.8 for all subcohorts and phases), and the trends observed in SG-adjusted and creatinine-adjusted OH-PAH concentrations were similar. Because of this and with the aim of achieving easier comparison with other studies that mostly published cratinine-adjusted OH-PAH concentrations, creatinine-adjusted values were used for further analysis. The SG-adjusted and non-adjusted values are, however, also reported.

The sum of PFAS concentration was calculated by summing the serum levels of the individual PFAS (including those which were below the LOD and their value was substituted by the value of LOD/√2). Similarly, the sum of all OH-PAH was calculated by summing the urine OH-PAH levels (including those which were below the LOD, and their value was substituted by the value of LOD/√2). Also, OH-PAH were summed by their native PAH (∑Naph (1-OH-Naph + 2-OH-Naph), ∑Fluo (2-OH-Fluo + 3-OH-Fluo), ∑Phen (2/3-OH-Phen + 9-OH-Phen + 1-OH-Phen + 4-OH-Phen), and 1-OH-Pyr).

Only OH-PAH and PFAS with >80 % of measurements above the LOD were selected for further analysis.

Principal component analysis (PCA) was used to explore the relationship between OH-PAH and PFAS levels and the distribution around axes of professional firefighters, new trainees (in all phases), and controls according to the chemical compounds. OH-PAH were summed as ∑Naph (1-OH-Naph + 2-OH-Naph), ∑Fluo (2-OH-Fluo + 3-OH-Fluo), ∑Phen (2/3-OH-Phen + 9-OH-Phen + 1-OH-Phen + 4-OH-Phen), and 1-OH-Pyr. Due to the log-normal distribution of concentrations, the compounds were log-transformed before analysis.

The potential association of lifestyle factors on the exposure of professional firefighters, new trainees, and controls to OH-PAH and PFAS, were tested. For this, information collected in exposure questionnaires was used (such as age, BMI, population size, former smoking habit, dental floss use, consumption of specific food supplements, participation in skiing, blood donation, use of GoreTex clothes/boot, type of heating and consumption of different food products (Table SI17)). Also, occupational factors like length of firefighting career, use of firefighting foam, and contact with a large fire in the last 6 months were tested (only for firefighters).

Nonparametric Spearman‘s correlation was used to determine the relationships between continuous exposure factors (specifically age, BMI, and population size), and urine levels of individual and summed OH-PAH and serum levels of PFAS separately for professional firefighters, new trainees, and controls in phase 1 (before training). Correlations were performed also for all firefighters (professional and new trainees together) in phase 1, where career length was also included.

For understanding the effect of categorical exposure factors, nonparametric Kruskal-Wallis ANOVA (KW test) and Mann-Whitney *U* test (MW test) were used to test for differences in concentrations of PFAS and OH-PAH. The non-parametric tests were used due to the non-homogeneity of the variances and non-equal sample sizes in some categories of exposure factors. As in the case of correlation, tests were performed separately for professional firefighters, new trainees, and controls and together for firefighters (professional and new) in phase 1 (before training). The test was performed if the number of participants in the exposure factor category was >10. KW was used for factors with multiple categories such as frequencies of consumption of particular food products while MW test was used for questionnaire parameters with two categories (YES/NO) such as former smoking habit, dental floss use, consumption of specific food supplements, participation in skiing, blood donation, use of GoreTex clothes/boots, use of firefighting foam, contact with fire, and type of heating. Effects of the use of firefighting foam and contact with a large fire in the last 6 months were tested only for firefighters (both professionals and new trainees).

KW test was also used to test for differences in levels of individual PFAS and OH-PAH among professional firefighters, new trainees, and controls, and between study phases.

Multivariate linear regression was used to explore the occupational and lifestyle factors associated with exposure of firefighters (professional firefighters and new trainees in phase 1) to selected PFAS (specifically PFOS, PFOA, PFNA, PFHxS) and OH-PAH (∑Naph, ∑Fluo, ∑Phen and 1-OH-Pyr). Exposure factors that were significantly associated with the individual or summed PFAS or OH-PAH levels from previous analyses (a significant correlation >0.1 for continuous variables, a significant difference in MW or KW test) were included in the regression analysis.

Concentrations were transformed by natural logarithm, and outliers were excluded before analysis. The normal distribution of residuals was checked by using histograms and the Kolmogorov-Smirnov test. Regressions for each exposure factor and selected PFAS and OH-PAH were adjusted for other exposure factors to explain the main effects of each factor. A regression model for each selected PFAS and OH-PAH was computed with all significant exposure factors to determine the maximum variability (coefficient of determination, R^2^) in PFAS and OH-PAH concentrations explained by the given factors.

Additionally, Ward's hierarchical clustering was performed as an explanatory technique for cumulative PFAS and OH-PAH exposure to determine the similarity of participants with respect to the exposure factors (questionnaire variables). All non-binary responses from the questionnaires were first converted to binary data (1/0), and a metric based on the Jaccard coefficient for binary data was used in the clustering. Then, serum PFAS levels and urine OH-PAH levels were compared among clusters using the Mann-Whitney *U* test. A detailed description of the method is in Supplementary material.

Data analyses and visualization were performed using MS Excel (version 2108), R Studio using R version 4.1.0, and ggplot2 package (Wickham, 2016) Origin 2021b, and Statistica (version 13.5.0.17).

## 3 Results

The limits of detection (LOD), and the detection frequencies (DF) of all analyzed compounds in all samples, as well as for each subcohort and phase, are presented in Tables SI08 (PFAS) and SI09 (OH-PAH). PFOA, PFNA, PFDA, PFHxS, and PFOS were detected with a frequency higher than 95 %. Other frequently detected were PFUnDA (94.4 %) and PFHpS (84.5 %). 1-OH-naphthalene, 2-OH-naphthalene, 2-OH-fluorene, and 2/3-OH-phenanthrene were the most detected OH-PAH in all samples (100 % DF). 1-OH-phenanthrene, 4-OH-phenanthrene, and 9-OH-phenanthrene were detected with frequency of 88.3 %, 92.6 %,and 47.3 %, respectively. 3-OH-benzo[*a*]pyrene was only detected in 1.3 % of samples (all in new trainees or professionals). Note that the LOD of 9-OH-phenanthrene and 3-OH-benzo[*a*]pyrene is 10-fold higher than of other OH-PAH, which might have affected their detection frequency.

### 3.1 Serum PFAS levels

**Fig. 2:**
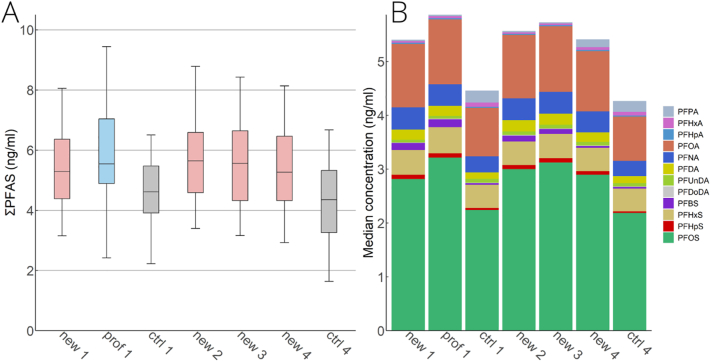
Serum ΣPFAS levels (ng/ml) in each subcohort and phase. (A) Median (line), 25th – 75th (box) and 5th – 95th (whiskers) percentiles. (B) The PFAS profile - the proportion of the median of each PFAS to the total PFAS median concentration in each subcohort and phase. New = new trainees, prof = professionals, ctrl = control group, the number represents the phase. Serum ΣPFAS levels between firefighters (new trainees and the professionals) and the controls were significantly different, though they did not differ significantly among the firefighters (professionals and new trainees) in any subcohort nor phase.

Firefighters (both new trainees and professionals) had 1.1–1.4 times higher total PFAS concentrations compared to the controls in all phases (Fig. 2A). Specifically, significantly higher concentrations (p < 0.05) were found for PFOA, PFNA, PFDA, and PFOS; however, no statistically significant difference was observed between serum ΣPFAS levels in new trainees and professionals in any of the phases. No statistically significant difference (p < 0.05) was observed for PFUnDA and PFHxS between any of the subcohorts and phases.

The PFAS profile (proportion of each PFAS to total PFAS) in the controls and the firefighters was comparable (Fig. 2B). In all subcohorts and phases, the PFAS with the highest medians was (listed by decreasing median concentration) PFOS, PFOA, PFHxS, and PFNA (Fig. SI10 and Table SI11), followed by PFDA for new trainees and professional firefighters, and PFPA for the control group.

### 3.2 Urine OH-PAH levels

Creatinine-adjusted urine OH-PAH levels were used for further statistical analyses. The non-adjusted and specific gravity-adjusted OH-PAH levels are presented in the Supplementary material (SI12 – SI14).

**Fig. 3:**
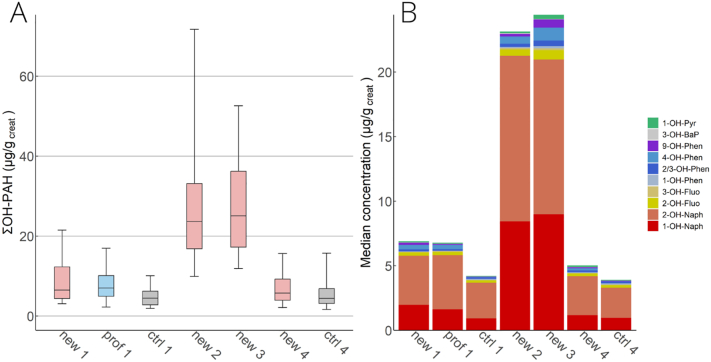
Urine OH-PAH levels in each subcohort and phase. (A) Box and whisker plots of the urine creatinine-adjusted sum of all measured OH-PAH concentrations (μg/g_creat_). Median (line), 25th–75th (box) and 5th – 95th (whiskers) percentiles. (B) The PAH profile (proportion of the median of each OH-PAH to the median ΣOH-PAH) in each subcohort and phase (B). New = new trainees, ctrl = control group, prof = professionals, the number represents the phase. Urine PAH levels in new trainees in phases 2 and 3 were significantly higher than in new, prof, and ctrl in phases 1 and 4. No significant difference in ΣPAH levels was observed between new trainees in phases 2 and 3 and between new, prof, and ctrl groups in phases 1 and 4.

The highest median urine ∑OH-PAH concentrations observed in new trainees 1 and 4 h after training with burning pallets (phases 2 and 3) were 3.4–5.7 times and significantly (p < 0.05) higher than the median concentrations in other subcohorts and phases (Fig. 3A, Table SI14, Fig. SI15). The concentrations of ∑OH-PAH in new trainees between phases 2 and 3 were not significantly different. It is noteworthy that for some participants, the peak ∑OH-PAH level was in phase 2 followed by a decrease in phase 3, while for some the peak PAH level was in phase 3 (Fig. SI16). ∑OH-PAH concentrations in professional firefighters, new trainees in phases 1 and 4, and the control group were not significantly different. This leads to the conclusion that firefighter training significantly increases OH-PAH concentrations within a few hours after the training, but within 4 weeks the concentrations return to their pre-training levels or levels in controls.

The PAH profile (the proportion of each OH-PAH to total OH-PAH) was similar in professional firefighters, new trainees, and controls (Fig. 3B). The exception was for new trainees in phases 2 and 3, where a greater proportion of 1-OH-Naph was observed compared to the other phases, suggesting that naphthalene contributes to the overall PAH exposure during training.

### 3.3 Overall exposure pattern

**Fig. 4:**
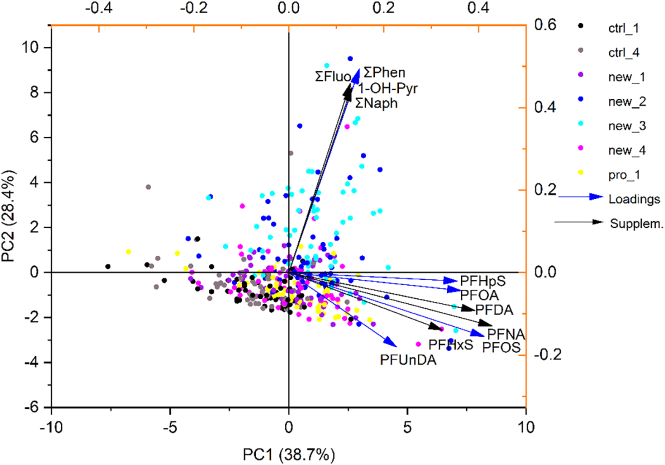
PCA for logarithmic levels of serum PFAS and PAH in urine (calculated as the sum of corresponding metabolites (OH-PAH)) in each subcohort and phase. Black arrows indicate the supplementary variables (variables were projected onto PCA axes but were not used for the PCA calculation).

Principal component analysis (PCA) showed that 80 % of individual PFAS were correlated (Fig. 4). Strong correlations were also observed among individual OH-PAH in all subcohorts, especially in new trainees and professionals. There were no or only weak inter-compound correlations between PFAS and OH-PAH in firefighters (professional and new trainees), while inter-compound correlations were observed in the control group. This indicates common exposure pathways for PFAS and PAH in firefighters and heterogeneous pathways in the control group. The correlation matrices for professional firefighters, new trainees, and controls are presented in the Supplementary material (Figs. SI04 – SI07).

PFAS are distributed along the first axis and represented by participants from both firefighters and controls. Firefighters and controls overlap, meaning that there are individual controls with increased PFAS levels and individual firefighters with decreased levels, but, in general, higher PFAS concentrations were found in firefighters.

PAH are distributed along the second axis, represented by the participants with lower PAH levels from all subcohorts and phases. In contrast, PAH levels in new trainees after training with burning wooden pallets (phases 2 a 3) were significantly elevated compared to controls and professional firefighters.

### 3.4 Comparison of PFAS and 1-OH-Pyr levels with reference values

**Fig. 5:**
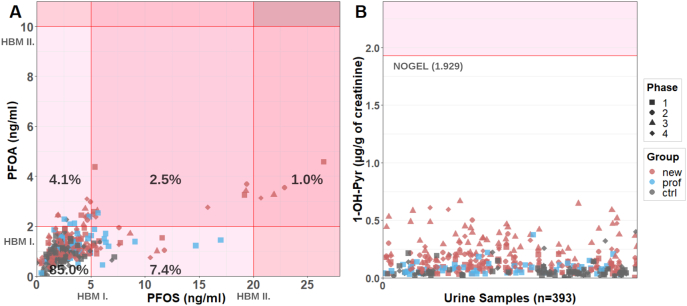
(A) Comparison of PFOS and PFOA levels in all samples (for all participants in each subcohort and phase, N = 394) with the HBM-I and HBM-II values. New = new trainees, prof = professional firefighters, and ctrl = control group. Exceeding the HBM-I value does not necessarily have to result in adverse health effects, but further monitoring is needed. The levels above the HBM-II value may result in adverse health effects. (B) The levels of urinary OH-Pyr in all participants and phases (N = 394) were compared to the “No observed genotoxic effect level in the body” (NOGEL) proposed by Jongeneelen (2014).

Serum PFOA and PFOS levels were compared with available HMB-I and HBM-II human plasma PFOA and PFOS values. For PFOS and PFOA, the HMB-I value in human blood plasma is set to 5 ng/ml and 2 ng/ml, respectively (Hölzer et al., 2021). HBM-II values were set to 10 and 20 ng/ml, for PFOA and PFOS, respectively. In our study, 10.9 % and 7.6 % of samples exceeded the HBM-I value for PFOS and PFOA, respectively (Fig. 5A). 3.5 % of measurements exceeded the HBM-I value for both compounds at the same time (Fig. 5A).

Urinary levels of 1-OH-Pyr in our participants were compared with the “No observed genotoxic effect level in the body” (NOGEL) proposed by Jongeneelen (2014). None of the measurements exceeded this value (Fig. 5B).

### 3.5 Factors associated with internal PFAS and PAH exposure

A summary of questionnaire answers is presented in Table SI17. Factors associated with internal exposure to individual PFAS and OH-PAH in phase 1 were tested separately for professionals, new trainees, the control group separately, and also for all firefighters (professionals and new trainees). Spearman's correlation was used for continuous exposure factors (Fig. SI04-SI07) and KW and MW tests for categorical factors.

### 3.6 Lifestyle factors

The effect of voluntary blood donation (in the hospital transfusion center) of participants on their PFAS levels was studied. 20.3 %, 18.2 %, and 28.9 % of new trainees, controls, and professionals, respectively, donated blood in the last 12 months before the study (Table SI17). PFOA, PFNA, PFOS, and PFHpS levels in blood donors among professional firefighters were significantly lower (MW test, p < 0.05) than in non-donors (Fig. SI18). No statistically significant differences in PFAS levels were observed in the controls and new trainees when they were divided into subsets according to their information on blood donation (yes/no) (MW test, p < 0.05). However, the statistical insignificance may be due to the small number of participants who donated blood (12 for new trainees and 10 for controls).

A weak but significant negative correlation (r_sp_ = 0.27–0.38) between population size and ΣFlu levels was observed in new trainees and the control group, and between population size and PFUnDA and PFDA (r_sp_ = 0.3–0.39) in new trainees. In general, the controls resided in areas with a higher median population size than the new trainees and the professionals. Also, higher OH-PAH concentration was observed in participants using coal or biomass for heating, however, this difference cannot be statistically proven due to the small number of samples in those categories.

BMI weakly but significantly positively correlated with 1-OH-Pyr (r_sp_ = 0.3) and 1-OH-Phen (r_sp_ = 0.3) in professional firefighters. In new trainees, no correlation of BMI with OH-PAH was observed; their BMI was, however, positively and significantly correlated with PFHpS (r_sp_ = 0.3). In the control group, BMI did not correlate with either OH-PAH or PFAS.

No significant differences in PFAS or OH-PAH concentration for other lifestyle factors such as former smoking habit, dental floss use, consumption of specific food supplements, participation in skiing, and use of GoreTex clothes/boots (MW test), as well as consumption of different food products including fish, smoked, fried or grilled food (KW test) were found (Supplementary material, section Lifestyle factors and exposure).

### 3.7 Occupational factors

PFAS levels and the length of firefighting career were weakly but significantly correlated (r_sp_ = 0.33). Similarly, some PFAS levels were positively correlated with age, especially in the control group (r_sp_ = 0.4–0.5), in which age was equally distributed across the whole age range.

A positive weak but significant correlation was also observed between age and 1-OH-Phen level (r_sp_ = 0.3), and between career length and 1-OH-Phen (r_sp_ = 0.5) and 1-OH-Pyr (r_sp_ = 0.3) in professional firefighters. No significant correlation was found between age and OH-PAH in new firefighters in training. In the control group, there was a weak but significant correlation between age, ΣPhen (r_sp_ = 0.3), 1-OH-Naph (r_sp_ = 0.3), and 1-OH-Pyr (r_sp_ = 0.4).

Professional firefighters were slightly, but significantly (KW test, p < 0.05) older than new trainees (Table 1). Their age positively correlated with the length of their firefighting career. This agrees with the fact that professionals were older and had been working longer as firefighters than the new trainees. Both, age and career length were associated with PFAS and some OH-PAH levels in firefighters, but because of their cross-correlation (r_sp_ = 0.6), the contribution of individual factors could not be unambiguously determined. Statistically significantly higher concentrations of ∑Naph, ∑Fluo, ∑Phen, and 1OHPyr were found in firefighters (KW test, p < 0.05) who had been in contact with larger fires in the past six months. There was no statistically significant difference in PFAS concentrations in firefighters (KW test, p < 0.05) who were in contact with firefighting foams in the last year.

Additionally, cluster analysis as an explanatory technique to determine the possible cumulative influence of all factors on PFAS and PAH levels was used. None of the clusters showed significantly different levels of PAH or PFAS. The method and results of the cluster analysis are described in the Supplementary material (Section Cluster analysis).

Occupational exposure might be the main driver of exposure to PFAS in new firefighters and professionals. Therefore, regression analysis was performed for the subset of new firefighters in training and professionals in phase 1 for four selected PFAS with the highest concentration contribution to serum levels and with the highest detection frequency (> 95 %), specifically PFOS, PFOA, PFNA, and PFHxS. In addition to occupational factors (length of firefighting career and contact with firefighting foam in the last year), other factors were added to the analysis, according to the results from the nonparametric tests and correlation analysis, namely blood donation in the last 12 months and population size in place of residence.

Regression analysis shows that of the factors included in the analysis length of firefighting career and blood donation in the last 12 months significantly contributed to firefighters' PFAS exposure (Table 2). In contrast, one use of firefighting foam had no significant effect on PFAS levels, while population size only had a significant effect on PFNA. Regression models which included all significant factors explained around 10 % of the variability of the levels of each of the four selected PFAS. The regression analysis confirmed the results of non-parametric tests and showed that firefighters with a longer firefighting career had higher serum PFAS levels, while donating blood decreased serum PFAS levels.

**Table 2:**
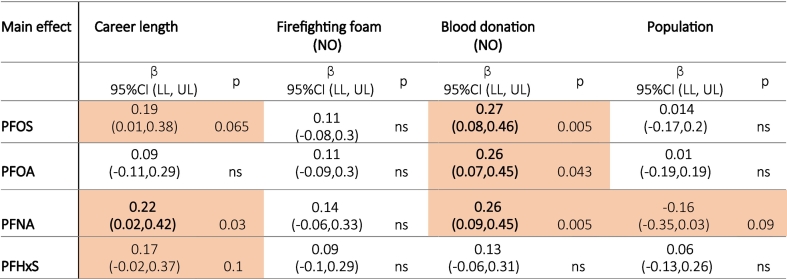
Regression analysis results for firefighters (professionals and new trainees, N = 111) in the first phase (regression coefficients and standard error SE), the relationships between each exposure factor (length of firefighting career, contact with firefighting foam in the last year (YES N = 38/NO N = 73), blood donation in the last 12 months (YES N = 29 / NO N = 82), and population size in place of residence) and selected PFAS were adjusted for other factors to explain the main effects of each factor. Orange color means results significant at p < 0.1; results significant at p ≤ 0.05 are shown in bold; CI: Confidence interval, LL: lower limit, UL: upper limit.

Additionally, regression analysis was also performed on the same subset (firefighters in training and professionals in phase 1) for ∑Naph, ∑Fluo, ∑Phen, and 1OHPyr for factors (age, BMI, population size, and contact with fire in the past 6 months) that were significantly associated with any of the OH-PAH in correlation or nonparametric tests. However, none of the regression models were statistically significant.

## 4 Discussion

### 4.1 PFAS exposure

Firefighters (new trainees already since the first phase, and the professionals), have significantly higher serum PFAS levels than the control group. The fire rescue service of the Czech Republic mainly assists with residential, commercial, industrial building, and vehicle fires. Firefighting foams might be one of the main sources of firefighters' exposure to PFAS. Firefighting foams used during the training and in the response to incidents in the Czech Republic at the time of the sampling (eg. Finiflam A3F/A, Sthamex F15 (3 %), and Fomtec MB5 (2–6 %)) contain various levels of the 12 PFAS analyzed in our study. The PFAS content might differ among the products. Also, due to the persistence of PFAS, contamination of the working/training environment due to the past use of firefighting foams containing PFAS might play a significant role in the PFAS exposure of firefighters. Other exposure pathways include firefighters' protective clothing, combustion of products containing the substances on the fire ground (e.g. electronics, furniture, stain-resistant carpeting, and insulation), or PFAS present in smoke while firefighting.

The increased PFAS levels in firefighters in comparison with the general population are in agreement with other studies, although the firefighters' PFAS levels observed in this study were lower than in American, Australian, and Finnish professional and voluntary firefighters (Nilsson et al., 2022a; Shaw et al., 2013; Dobraca et al., 2015b; Graber et al., 2021; Rotander et al., 2015; Laitinen et al., 2014). For example, PFOS, and PFHxS serum levels in Australian firefighters reported by Nilsson et al. (2022a) are approximately 4.5 and 13 times higher, respectively, than the levels in Czech firefighters. The serum PFOA level is, however, comparable between the Australian firefighters reported by Nilsson et al., and the Czech firefighters reported in this study. PFOA, PFOS, and PFHxS levels in the serum of US voluntary firefighters collected in 2019 and reported by Graber et al. (2021) are 1.7, 1.3, and 3.7 times higher than in this study.

Although PFAS levels in some firefighters were comparable to the control group, we observed generally increased serum PFAS levels in new trainees already in the first phase before training. This could be explained by the fact that some new trainees had been employed for several months in the firefighting service before training. Although they did not work with firefighting foam and did not participate in response to incidents, they spent time in the firefighters' working environment (vehicle cabins, training centers, fire stations), handled potentially contaminated personal protective equipment (PPE), or worked as voluntary firefighters in the past. Although the information on whether the new trainees in this study had previously been working as voluntary firefighters, is not available, and this is one of the study's limitations, it is highly probable. In the Czech Republic, there is high number of voluntary firefighters and many of them are often recruited for professional firefighting occupation.

The inter- and intra-compound group correlation patterns suggest common sources of exposure to PFAS and PAH among firefighters. One training with firefighting foams did not significantly increase PFAS levels; nor was any effect of firefighting foams use frequency observed, probably because the reported firefighting foams frequency was relatively low even among professional firefighters (25 % of professionals had not been exposed to firefighting foams in the previous year and 34.6 % were exposed two or more times). The positive correlation of career length or age with PFAS levels is in agreement with the fact that PFAS can accumulate in the body due to their medium to long elimination half-lives (Xu et al., 2020). But the effect of firefighting foams, firefighting career length (and non-occupational factors like population size, and blood donation) only explained approximately 10 % of the variability of PFAS levels according to the regression analysis. The increased PFAS levels in firefighters, therefore, seem to be the result of multiple exposures to different sources and at different frequencies, which might even be individual-specific.

Serum PFOA and PFOS levels were compared with available HMB-I and HBM-II human plasma PFOA and PFOS values. These values, derived by the HBM Commission, are based on human epidemiological studies. Several adverse health effects including fertility and pregnancy, birth weights, lipid metabolism, immunity, hormonal development, thyroid metabolism, and onset of menopause were evaluated to derive the HBM values (Ganzleben et al., 2017; Hölzer et al., 2021; Schümann et al., 2021). The HBM-I value for a given substance is the concentration of that substance in human biological material, below which no adverse effects on human health are expected. Such values are derived for preventive healthcare, and if not exceeded, no action is required (Hölzer et al., 2021). For PFOS and PFOA, the HMB-I value in human blood plasma is set to 5 ng/ml and 2 ng/ml, respectively (Hölzer et al., 2021). The HBM-II value is a threshold above which exposure will almost certainly lead to adverse health effects, and there is a need for exposure reduction based on biomedical advice (Ganzleben et al., 2017; Hölzer et al., 2021). HBM-II values were set to 10 and 20 ng/ml, for PFOA and PFOS, respectively.

In our study, 10.9 % and 7.6 % of samples exceeded the HBM-I value for PFOS and PFOA, respectively (Fig. 5A). 3.5 % of measurements exceeded the HBM-I value for both compounds at the same time. Most of the measurements that exceeded the HBM-I values for PFOS and/or PFOA came from new trainees and professionals, but a few of them also from the controls. Exceeding the HBM-I value does not necessarily mean that participants would experience adverse health effects, but detailed monitoring and prevention towards exposure should be required (Hölzer et al., 2021; HBM-Commission, 2000). The HBM-II value was exceeded in 1 % of our measurements (Fig. 5A) for both PFOA and PFOS at the same time, all of these measurements corresponding to the same participant (new trainee) in all study phases. Firefighters' exposure to PFAS is associated with several health markers, for example, DNA methylation, epigenetic age, and its acceleration, cardiometabolic, kidney, or thyroid markers (Nilsson et al., 2022b; Goodrich et al., 2021). When the HBM-II value is exceeded, control measurement is recommended and then the identification and elimination of possible sources of the exposure of the individual should be carried out. These might include occupational sources as well as contaminated food and drinking water (Schümann et al., 2021).

### 4.2 PAH exposure

The observation of increased urine OH-PAH levels after combustion training is in accordance with other studies. Banks et al. (2021) observed an increase in OH-Naph, OH-Fluo, and OH-Phen in participants who attended particleboard fires, which were characterized by a high concentration of PAH in the smoke layer. Fent et al. (2019) observed a significant increase in the concentration of OH-PAH in urine 3 h after the training of firefighters in different combustion scenarios (bravo OSB, alpha OSB, pallet and straw, simulated smoke). Laitinen et al. (2010) observed elevated urine concentrations of 1-OH-Naph and 1-OH-Pyr after training with burning materials. Although the OH-PAH levels in new trainees increased significantly 1 h and 4 h after the training with burning pallets, they returned to their pre-training levels or levels in controls within 4 weeks. That is in agreement with the fact that OH-PAH elimination occurs within several hours after exposure (Motorykin et al., 2015; Lutier et al., 2016).

Comparison with OH-PAH levels in firefighters revealed in other studies is challenging because different designs (sources of PAH, sampling time since exposure, exposure scenarios), as well as compounds, are reported. We compared our results with those of Banks et al. (2021), who measured urinary OH-PAH in Australian firefighters several hours after training activities. Although the study design was not identical, we found it largely similar to ours. The levels of ΣNaph were two- to three-times higher than the medians in Banks et al. (median 8.3 and 8.5 μg/g_creat_ 1.9 and 4.6 h after training, respectively). For 1-OH-Pyr, our levels in phase 2 were comparable with the median in Banks et al. (0.14 μg/g_creat_), but the levels 4 h after training were >2-times higher than in Banks et al. 4.6 h after combustion training (0.14 μg/g_creat_). Even though study designs may be similar, it is not always possible to extrapolate the findings from one study to another, especially not for compounds with fast elimination and therefore high temporal variability in internal levels (Calafat, 2016; Aylward et al., 2014), or mixtures whose compositions vary according to the source.

With the exception of training with burning pallets and contact with fire in the past six months, none of the studied factors seemed to significantly influence PAH levels, probably also due to their fast (within a few hours) elimination (Huang et al., 2007; Jongeneelen et al., 1990; Brzeźnicki et al., 1997). The correlation between age and some OH-PAH levels observed in professional firefighters and the controls might reflect different metabolism rates, lifestyles, and dietary habits (Yang et al., 2021) rather than substantial bioaccumulation. Although the type of heating in participants' homes was not statistically significant for PAH levels, a weak negative correlation between PAH and population size in the place of residence was observed. It is possible that the greater tendency to use biomass or coal for heating in smaller population centres, and especially in the heating season, might influence air quality and the presence of PAH in the respective environment (Sykorova et al., 2015).

HBM-I and HMB-II values have not been established for PAH. Reference values for the general population or occupationally exposed workers were derived for some OH-PAH (Khoury et al., 2018; Schulz et al., 2011; Unwin et al., 2006; Boogaard, 2007) but unlike HBM-I and -II values, these reference values are not health-related. Due to the carcinogenicity of PAHs, setting a no-risk level is problematic. Therefore, in the occupational environment, the acceptable/tolerable cancer risk level approach is often used to set exposure limits for carcinogens. 1-OH-pyrene is often used as a surrogate biomarker of PAH exposure because it is always present in PAH mixtures (Jongeneelen, 2014). A Risk-based limit value has not been established for 1-OH-pyrene, due to the lack of human/animal epidemiological studies that would link exposure with cancer. However, Jongeneelen (2014) proposed an alternative method of setting a health-based limit for 1-OH-pyrene. On the basis of a metaanalysis of studies linking urinary levels of 1-OH-pyrene and early genotoxic effects, they proposed a No Observed Genotoxic Effect Level (NOGEL) value of 1.0 μmol/mol_creat_ (1.929 μg/g_creat_) for 1-OH-Pyr in urine at the end of the shift (Jongeneelen, 2014).

We compared the urinary levels of our participants with the NOGEL set by Jongeneelen (2014) (Fig. 5B). None of the measurements exceeded this value. It should be noted that the value determined by Jongeneelen et al. and its use in our study has the following limitations. i) Pyrene is not carcinogenic and the composition of PAH mixtures can vary depending on the source and setting; therefore, information on the pyrene/B[*a*]P ratio is important to understand the risks. The key studies from which the value by Jongeneelen et al. was determined were performed on coke-oven workers. It is possible the PAH mixture composition in our study varied from the mixture in those studies.(Jongeneelen, 2014) ii) The value determined by Jongeneelen et al. does not take into account life-long exposure. iii) Due to the nature of firefighting activities, the PAH mixtures to which firefighters are exposed can differ from those in the environment of coke-oven workers.

Considering the carcinogenic potential of PAH and the potential variation of PAH mixtures depending on the source and setting, an accurate assessment of the risks to firefighters posed by exposure to PAH based merely on the monitoring of internal PAH levels and comparing them with the guidance value for 1-OH-Pyr does not seem feasible. Parallel genotoxicity effect testing would provide better information on the link between PAH urinary levels and genotoxic effects in firefighters. Methods such as the comet assay offer a fast and relatively cheap way to assess the early genotoxic effects of environmental exposure and are feasible for use in human biomonitoring studies (Anderson et al., 2013).

### 4.3 Strengths and limitations

The CELSPAC – FIREexpo study is unique in Central and Eastern Europe because of its complexity, extent, and long-term design. The study combines monitoring with basic research, and therefore its findings have implications that are not restricted only to the firefighting profession in the Czech Republic. Aliquots of the tissues used in this research are saved in the CELSPAC Biobank facility, enabling further analyses and research. Most of the participants expressed their informed consent to allow the data collected in this study to be linked with national health registers, and to be contacted again for longitudinal monitoring or follow-up studies.

The limitations of the study include a lack of certain information that would help to elucidate some exposure sources, such as information about the previous employment of new trainees, more detailed information on factors contributing to PAH exposure, and data from exposure questionnaires that would regard other types of chemical compounds (the exposure questionnaires were focused on PFAS and PAH). Also, when the participants are subset into categories according to the information in questionnaires, the number of participants in each category is rather low, which can bring a certain degree of uncertainty when concluding the impact of individual factors on the internal levels of chemicals. The study participants were all male, because, until recently, there were no female professional firefighters actively participating in responses to incidents. This has now changed, as, in August 2022, the first women began to qualify for active participation in responses to incidents. Thus, we expect that more women will now become active professional firefighters, and that, therefore, the collection of data on women participants will be necessary.

## 5 Conclusions

The firefighting occupation and firefighting training lead to increased PFAS and PAH levels in firefighters. Health-based HBM-I and HBM-II values for PFOS and PFOA were exceeded in several participants; thus, there is a need to minimize such exposure and to conduct further monitoring. Although there might be common sources of exposure to PAH and PFAS in firefighters, no single factor or source was shown to predominate; rather, the PFAS and PAH levels measured in this study appeared to result from a combination of multiple sources and exposure frequencies.

The findings of our study highlight the importance of monitoring chemical exposure and associated risks in firefighters. We propose the following recommendations:

Establish the long-term monitoring of internal levels of priority chemicals in firefighters.

Identify the dominant sources of exposure to PAH and PFAS and take steps to eliminate or minimize them.

Keep a record of the number, frequency, and nature of incidents faced by individual firefighters in order to manage their occupational activities relating to chronic exposure to hazardous chemicals and its health risks.

Simultaneously monitor the internal levels of contaminants and their biological effects, especially with respect to PAH mixtures that vary in time and in different settings, in order to better understand the risks resulting from exposure. Such assessment should also include information on individual susceptibility to the effects of exposure.

Provide information to firefighters on the risks of chronic occupational exposure to PFAS and PAH and how to minimize them through safety training.

Decontaminate PPE and the occupational environment to reduce the accumulation of hazardous chemicals, which, over time, may cause adverse health affects.

## Ethics approval

The study was approved by the ELSPAC Ethics Committee, ethical approval number No: ELSPAC/EK/1/2019. All participants received an information brochure and participated in personal interviews to be fully informed about the study and their participation, and informed consent was obtained from each participant before participation in the study. All data were pseudonymized to protect the identity of the participants.

## Funding

This project received funding under grant agreement No. 857340 and No. 874627 and was supported under grant agreement No. 857560 from the European Union's 10.13039/100010661Horizon 2020 research and innovation program. This work was carried out in the framework of the European Partnership for the Assessment of Risks from Chemicals (PARC) and has received funding from the 10.13039/501100000780European Union's Horizon Europe research and innovation programme under grant agreement No. 101057014. This publication reflects only the authors' views and the European Commission is not responsible for any use that may be made of the information it contains. This work was also supported by Cetocoen Plus project (CZ.02.1.01/0.0/0.0/15_003/0000469). The authors also thank specific research infrastructure MUNI/A/1135/2018 (ID=46868) and MUNI/A/1032/2019 (ID=54811) for supportive background.

## CRediT authorship contribution statement

**Katarína Řiháčková**: Conceptualization, Methodology, Validation, Formal analysis, Investigation, Data curation, Writing – Original Draft & Review and Editing, Visualization.

**Aleš Pindur:** Conceptualization, Methodology, Validation, Investigation, Resources, Writing – Review and Editing, Project administration, Funding acquisition.

**Klára Komprdová:** Formal analysis, Investigation, Visualization, Data curation, Writing – Original draft & Review and Editing.

**Nina Pálešová:** Formal analysis, Visualization, Writing – Review and Editing.

**Jiří Kohoutek**: Formal analysis, Writing - Original Draft & Review and Editing.

**Petr Šenk:** Formal analysis, Writing - Original Draft & Review and Editing.

**Jana Navrátilová:** Investigation, Writing – Review and Editing.

**Lenka Andrýsková**: Resources, Project Administration, Writing – Review and Editing.

**Ludmila Šebejová:** Formal analysis, Writing – Review and Editing.

**Richard Hůlek**: Data curation, Software, Writing – Review and Editing.

**Mazen Ismael**: Data curation, Software, Writing – Review and Editing.

**Pavel Čupr**: Conceptualization, Methodology, Investigation, Resources, Writing –Review and Editing, Funding acquisition, Supervision.

## Declaration of competing interest

The authors declare that they have no known competing financial interests or personal relationships that could have appeared to influence the work reported in this paper.

## Data Availability

Summary data from the CELSPAC – FIREexpo study are incorporated into the article and its online Supplementary material. Individual data are available on request and on the approval of the steering committee. The Population CENSUS data are owned by Czech Statistical Office (CZSO), and are publicly available at: https://www.czso.cz/csu/czso/pocet-obyvatel-v-obcich-za0wri436p. The addresses of participants and other sensitive data cannot be shared for ethical/privacy reasons.

## Appendix A Supplementary data

Supplementary material Image 1
