# Supplementary material for: The exposure of Czech firefighters to perfluoroalkyl substances and polycyclic aromatic hydrocarbons: CELSPAC – FIREexpo case-control human biomonitoring study
Source: Sci Total Environ. 2023 Jul 10;881:163298. doi: 10.1016/j.scitotenv.2023.163298 (PMC10230324; doi:10.1016/j.scitotenv.2023.163298)
Supplement: Supplementary file 1 — Supplementary material [file mmc1.docx]

Supplementary material for „The exposure of Czech firefighters to perfluoroalkyl substances and polycyclic aromatic hydrocarbons: CELSPAC – FIREexpo case-control human biomonitoring study“

Authors

Katarína Řiháčková^1^ (katarina.rihackova@recetox.muni.cz), Aleš Pindur^1,2,3^ (ales.pindur@svzbm.izscr.cz), Klára Komprdová^1^ (klara.komprdova@recetox.muni.cz), Nina Pálešová^1^ (nina.palesova@recetox.muni.cz), Jiří Kohoutek^1^ (jiri.kohoutek@recetox.muni.cz), Petr Šenk^1^ (petr.senk@recetox.muni.cz), Jana Navrátilová^1^ (jana.navratilova@recetox.muni.cz), Lenka Andrýsková^1^ (lenka.andryskova@recetox.muni.cz), Ludmila Šebejová^1^ (ludmila.sebejova@recetox.muni.cz), Richard Hůlek^1^ (richard.hulek@recetox.muni.cz), Mazen Ismael^1^ (mazen.ismael@recetox.muni.cz), Pavel Čupr^1^* (pavel.cupr@recetox.muni.cz)

^1^RECETOX, Faculty of Science, Masaryk University, Kamenice 753/5, 625 00 Brno, Czech Republic

^2^Faculty of Sports Studies, Masaryk University, Kamenice 753/5, 625 00 Brno, Czech Republic

^3^Training Center of Fire Rescue Service, General Directorate of Fire Rescue Service of the Czech Republic, Ministry of the Interior of the Czech Republic, Trnkova 85, 628 00 Brno, Czech Republic

Corresponding author

## *Pavel Čupr, RECETOX, Faculty of Science, Masaryk University, Kamenice 753/5, 625 00 Brno, Czech Republic, pavel.cupr@recetox.muni.czTraining with burning pallets and firefighting foams

The new trainees were instructed not to eat grilled food, vitamin supplements, and beetroot products, and to avoid proximity to fire three days before the training. The drinking regime of participants was controlled before, during, and after the training to ensure proper hydration and smooth urine and blood sampling. For drinking, non-sparkling water with low nitrate levels was used. The ion drinks were not used.

On the day of the training on combating the fire in an enclosed space, which relates to the 2^nd^ and 3^rd^ phases of the study, trainees had breakfast at 7 am, put on protective clothes at 8 am, received short instructions, and practiced the use of the fire hose nozzle. Then, at about 10 am, they started the training. It took place in the metal flashover container (Figure SI01) for solid fuels to simulate real fire conditions.

The combustion takes place in the combustion chamber, which is accessed through the loading space and the loading door. Through the loading door, the fuel for the combustion (wooden disposable pallets) is loaded and the fire is ignited and flared up. The loading door is then closed and remains closed throughout the training duration.

The training container is completely enclosed when the trainees and instructors enter its training area (2.5 x 2.5 x 12 m size), and the combustion chamber door is open to achieve the consumption of oxygen and the entering of the exhausts into the container. During this time, trainees observe the emergence of the fire and combustion products inside the container, and the development and lowering of the neutral plane.

Next, doors No. 1 and 2 and windows No. 1 and 2, and the chimney are successively opened and closed to simulate the impact of air (oxygen) supply in different phases of the burning process and accompanying phenomena, including a flue gas discharge. Also, the impact of oxygen supply from different distances from the ignition source is simulated and observed.

Then, the fire is suppressed by closing the combustion chamber door, while the ignition source is not extinguished. The flashover effect is induced by opening the combustion chamber door and door No. 3. The trainees attempt to suppress the fire and push it back to the combustion chamber.

During this training activity, the temperature inside the training container can range from 100 to 500 °C depending on the phases of burning regulated by the air supply. The trainees spend 45 – 50 minutes inside the container. Each trainee wears personal protective equipment, which consists of emergency clothing (approved to technical standards ENISO13688:2014, EN1149-5:2008, and ČSN EN 469:2015/A1:2015/Opr.: 1:2005), a protective helmet (approved to technical standards EN443:2008, EN14458, EN14458), emergency gloves (approved to technical standards EN 659:2003+A1:2008), emergency shoes ( approved to technical standards ČSN EN 345), and self-contained breathing apparatus (approved to technical standard EN137:2006, Type 2) with the protective mask (approved to technical standard EN 136 class 3). The training with firefighting foams took place on the 9th or 10th week of the training, 1-2 weeks before
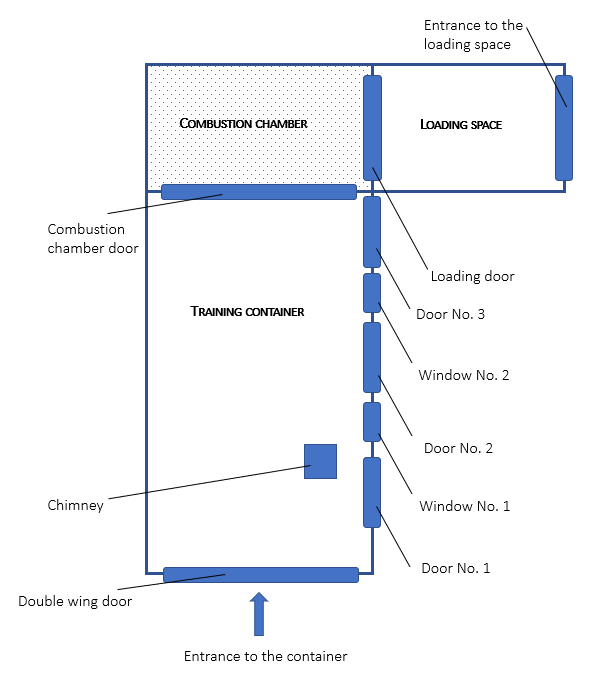
phase 4 of the study.

Figure SI01: Scheme of the training container

For the training with firefighting foams, which took place before the 4^th^ study phase, a simulator consisting of upper and lower baths with burners was used.

The baths of the simulator are filled with water, which is permeated by flammable gas. The fire incident is simulated by the ignition of the gas on the water's surface. The training consists of using different types of firefighting foams to extinguish the flammable liquids and to cool tanks to prevent liquids from the ignition.

The training took place in the open air. In the first phase of the training for approximately 30 minutes, participants use protective clothing against flame and radiant heat (ISOTEMP 2000, type 3). This clothing provides high-level protection, its use is, however, accompanied by an extreme physical and psychological burden to the firefighter. In the second training phase, participants are wearing standard emergency clothing, a safety helmet, protective helmet, emergency gloves, emergency shoes, and self-contained breathing apparatus (same as during the training in the enclosed space). The net training time of both phases together is in total approximately two hours. From 75 to 100 liters of firefighting foams are usually consumed during one training.

## Chemical analysis

### Urine OH-PAH^1^

Urine samples were heated to room temperature and homogenized using a vortex instrument, and 500 µl of each urine sample was transferred to a 96-well plate. 500 µl of β-glucuronidase (*E. coli* Type IX-A, 500U) solution and internal standards (1ng/sample each, except 2 hydroxynaphthalene, that was added in a concentration of 10ng/sample) in hydrolyzing buffer (0.1 M ammonium acetate, pH 6) were added into each well, and the content was mixed. The mixture was incubated for 2 hours at 55 °C and the reaction was stopped using 50 µl of 1 % acetic acid. Samples were then extracted using SPE plate Oasis HLB (60 mg). SPE conditioning was performed using 1 ml of methanol followed by 1 ml of water for equilibration. Samples were washed using 1 ml of 40 % methanol and eluted using 1.5 ml of acetone to the 96-well plate containing 10 µl of DMSO as a keeper. After evaporation to 10 µl of DMSO under nitrogen at 55 °C, the samples were diluted by  500 µl of 50 % methanol, homogenized, and analyzed on an LC-MS/MS system.

OH-PAH, the metabolites of PAH, namely 1‑hydroxynaphthalene (1‑OH‑Naph), 2‑hydroxynaphthalene (2‑OH‑Naph), 2‑hydroxyfluorene (2‑OH‑Fluo), 3‑hydroxyfluorene (3‑OH‑Fluo), 2/3‑hydroxyphenanthrene (2/3‑OH‑Phen), 9‑hydroxyphenanthrene (9‑OH‑Phen), 1‑hydroxyphenanthrene (1‑OH‑Phen), 4‑hydroxyphenanthrene (4‑OH‑Phen), 1‑hydroxypyrene (1‑OH‑Pyr), and 3‑hydroxybenzo[a]pyrene (3‑OH‑BaP), were analyzed using an Agilent 1200 series liquid chromatography (HPLC) system. Chromatographic separation was accomplished using a Waters Acquity BEH C-18 analytical column (100 x 2.1 mm, 1.7 μm particle size) maintained at 30 °C and equipped with an Acquity BEH C-18 VanGuard pre-column. The mobile phases for the gradient separation of the analytes were a 0.1 mM water solution of ammonium fluoride (component A) and methanol with the addition of 0.1 mM ammonium fluoride (component B). The flow rate was 0.3 ml/min, and the injection volume was 5 μl. Analyte detection was performed using an AB Sciex QTrap 5500 tandem mass spectrometer operating in negative electrospray ionization mode at 450 °C, with N_2_ as a nebulizer gas and a capillary voltage of -4kV. The 2D- and 13C-labeled isotope dilution method was used for the quantification of the analytes. The linear quantification range (MRM mode) was 0.1-100 µg/l urine, with limits of quantification from 0.018 to 0.2 µg/l urine for the respective analytes (MQL, calculated as 10‑times the standard deviation (SD) of the blank sample.

### Serum PFAS^2^

Serum samples were heated to room temperature and homogenized using a vortex instrument, and 200 µl of each serum sample was transferred to a 96-well plate (Phree Phospholipid Removal Plate - Phenomenex). 5 ng of Internal standard (isotopically labeled compounds) and 600 µl of acetonitrile with the addition of 1 % formic acid were added to each sample. Samples were mixed, filtered, moved to glass vials, and evaporated to the last drop of solvent under a nitrogen stream. 50 µl of methanol and 50 µl of ammonium acetate in water were added.

PFAS, namely perfluoropentanoic acid (PFPA), perfluorohexanoic acid (PFHxA), perfluoroheptanoic acid (PFHpA), perfluorooctanoic acid (PFOA), perfluorononanoic acid (PFNA), perfluorodecanoic acid (PFDA), perfluoroundecanoic acid (PFUnDA), perfluorododecanoic acid (PFDoDA), perfluorobutanoic acid (PFBA), perfluorohexane sulfonic acid (PFHxS), perfluoroheptane sulfonic acid (PFHpS), and perlfuorooctane sulfonate (PFOS), were analyzed using a QTrap 5500 LC-MS/MS system (ABSciex, CA, USA) with (ESI) equipped by a SYNERGI 4μ Fusion MAX-RP 80Ä 100 mm x 2 mm chromatographic column (Phenomenex, USA) with a Phenomenex SecurityGuard C18 4 x 2 mm pre‑column.

The mobile phases were methanol with 5 mM ammonium acetate in water 55:45 (component A) and methanol (component B). Gradient elution was used.

The value of LOD was determined from the calibration curve and signal of noise. The LOD for each analyzed PFAS is reported in Table SI08.

### Urine creatinine

Creatinine levels in urine were determined by LC-MS/MS using a modified procedure described by Dereziński et al.^3^ The Isotope dilution method was used for quantification of the analyte. The linear quantification range was 1-300 mg/dL, with limits of detection and quantification of 0.25 and 0.84 mg/dL, respectively. Seronorm™ Urine L-1 and L-2 certified reference materials were used periodically to verify the performance of the method.

The QA/QC for all chemical analyses is described in the Supplementary material. The method for determining specific gravity is described in the Supplementary material.

### Urine specific gravity

To determine the urine specific gravity (SG), a handheld refractometer Atago PAL-10S was used. The refractometer was blanked using 300 µL of MilliQ water, the refractometer was dried with a paper towel two times. The urine sample was vortexed and 100 µL of the urine sample was pipetted onto the refractometer. The specific gravity of the sample was recorded after the temperature had stabilized.

### QA/QC

Procedural blanks, as well as reference materials (NIST SRM 1957, 3672, 3673, and HBM4EU interlaboratory study reference sample), were processed as samples. Provided data are recovery and blank corrected. For each compound, the limit of quantitation (LOQ) was set as 3‑times standard deviations (SD) of blank values, and the limit of detection (LOD) as 1.5*SD. Reference samples were in good agreement with their certified or reference values.

Each batch of samples contained blank samples (matrix), reference material (pooled sample with known concentrations of analytes obtained during HBM4EU inter-calibration study), and spiked in-house QC sample (urine spiked with a known concentration of analytes.

Trace analytical laboratory is accredited under ČSN EN ISO/IEC 17025:2018 and was successful in all rounds of ICI/EQUAS European HBM4EU inter-calibration tests.

## Urine specific gravity and creatinine

Table SI02: Adjustment of urine OH-PAH concentrations for creatinine and specific gravity.

| To obtain the creatinine-adjusted OH-PAHs urine concentrations, Equation 1 was used.  Eq. 1:  $c_{{OH-PAHs}_{creat}}=\frac{c_{OH-PAHs}}{c_{creat}}$  where c_OH-PAHscreat_ is creatinine adjusted OH-PAHs urine concentration [µg_OH-PAHs_ /g_creatinine_, c_OH-PAHs_] is OH-PAHs concentration in urine [ng/ml], and c_creat_ is urine creatinine concentration [g/l]. |
| --- |
| For the adjustment of urine OH-PAHs concentrations for the urine specific gravity, the following equation (Equation 2) by Suavé et al.^4^ was used:  Eq. 2:  $C_{SG}= \frac{C_{i}\times({SG}_{ref}-1)}{{SG}_{meas}-1)}$  where C_SG_ is the concentration standardized on SG, C_i_ is the measured concentration, SG_meas_ is the measured specific gravity, and SG_ref_ is the reference SG value. As a reference SG value, a mean SG value for all cohorts was used (1.02). |

Table SI03 Descriptive statistics for the levels of urine creatinine (g/l) and specific gravity (unitless) in each subcohort and phase. New = new trainees, prof = professional firefighters, ctrl = control group. All creatinine levels were above the LOD (LOD = 0.25 µg/l). There is a statistically significant drop (Kruskal-Wallis ANOVA, p < 0.05) of urine creatinine concentration in the new trainees in phases 2 and 3 in comparison with new trainees in phases 1 and 4 and controls. This is in accordance with the fact that in new trainees in phases 2 and 3, the morning urine sampling was not possible due to the training schedule, and therefore the sampled urine was possibly more diluted (less creatinine, lower specific gravity). However, decreased creatinine concentration was also observed in professional firefighters where the morning urine was sampled. A similar trend was observed for the values of specific gravity.

| **Subcohort phase** |  | **Creatinine (g/l)** | **Specific gravity** |
| --- | --- | --- | --- |
| **new1**  **(N = 58)** | Median  Min - Max  10^th^ - 90^th^ perc. | 1.74  0.24 - 3.97  0.99 - 3.24 | 1.02  1 - 1.03  1.01 - 1.03 |
| **new2**  **(N = 58)** | Median  Min - Max  10^th^ - 90^th^ perc. | 0.88  0.21 - 3.52  0.27 - 2.05 | 1.01  1 - 1.03  1 - 1.02 |
| **new3**  **(N = 58)** | Median  Min - Max  10^th^ - 90^th^ perc. | 1.21  0.12 - 6.26  0.38 - 2.92 | 1.02  1 - 1.03  1.01 - 1.03 |
| **new4**  **(N = 58)** | Median  Min - Max  10^th^ - 90^th^ perc. | 1.81  0.79 - 3.91  1.22 - 3.21 | 1.02  1.01 - 1.03  1.01 - 1.03 |
| **prof1**  **(N = 52)** | Median  Min - Max  10^th^ - 90^th^ perc. | 1.37  0.45 - 3.24  0.83 - 2.14 | 1.02  1.01 - 1.04  1.01 - 1.03 |
| **ctrl1**  **(N = 55)** | Median  Min - Max  10^th^ - 90^th^ perc. | 2.14  0.66 - 5.58  1.05 - 3.95 | 1.02  1.01 - 1.04  1.02 - 1.03 |
| **ctrl4**  **(N = 54)** | Median  Min - Max  10^th^ - 90^th^ perc. | 2.14  0.51 - 5.11  0.77 - 3.67 | 1.02  1.01 - 1.04  1.01 - 1.03 |
|  |  |  |  |

## Spearman correlation of internal exposure, BMI, age, population, and length of the firefighting career


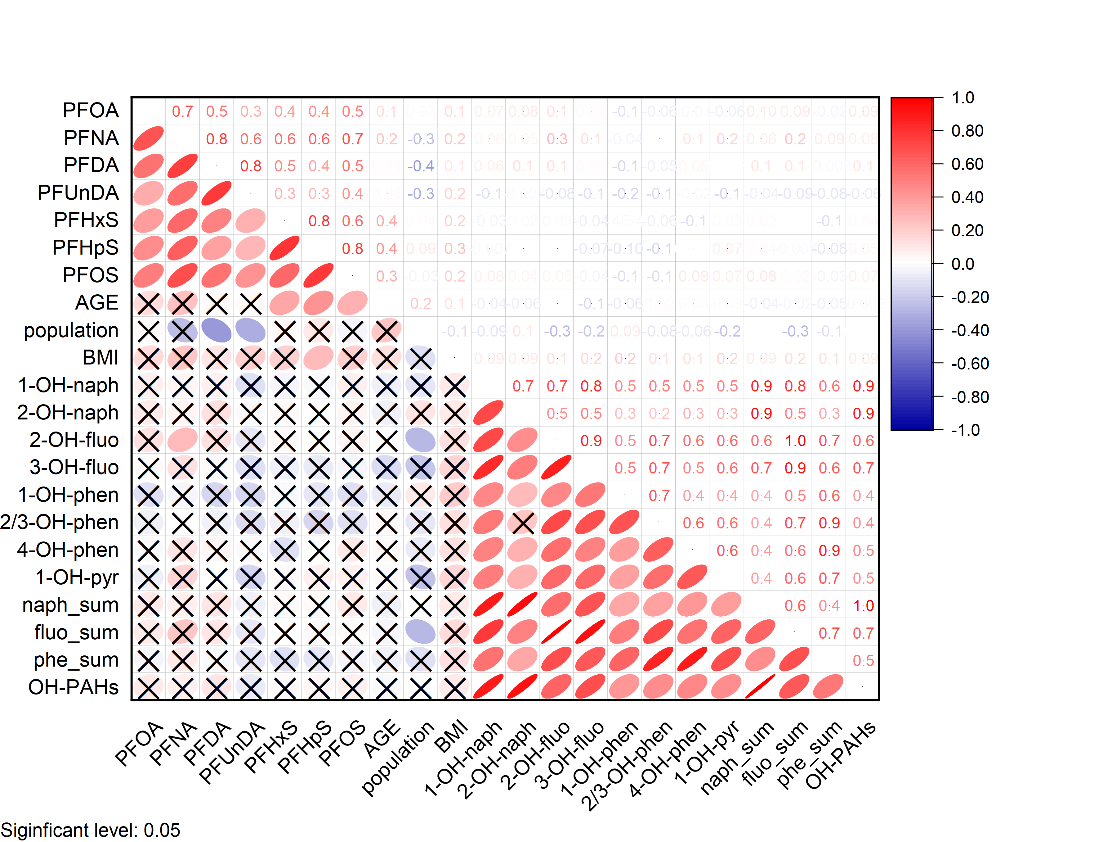


Figure SI04: Spearman’s correlation of serum PFAS concentrations, urinary OH-PAH concentrations, BMI, age, and population size in the new trainees in phase 1 (before training). Here, the parameter career length was not used due to the low variance of the career length in new trainees. Significance level = 0.05. The non-significant correlations are marked with a cross.


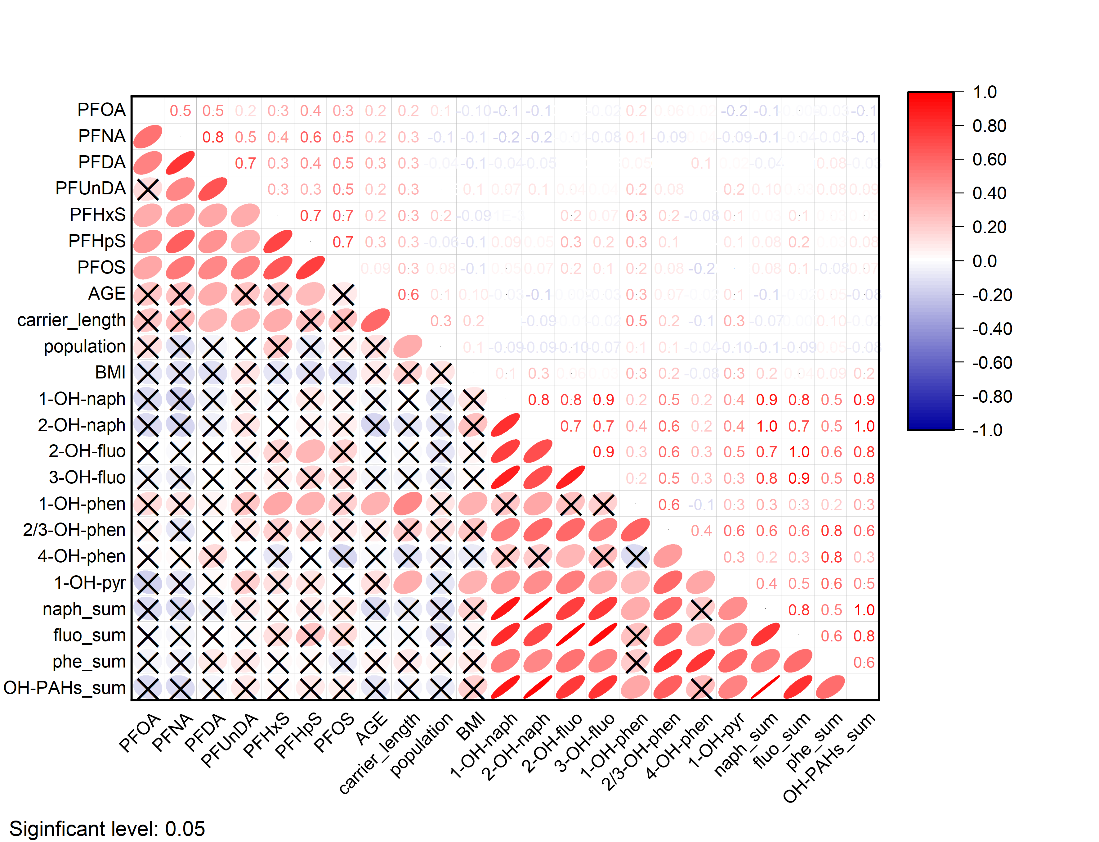


Figure SI05: Spearman’s correlation of serum PFAS concentrations, urinary OH-PAH concentrations, BMI, age, population size, and length of firefighting career in the professional firefighters in phase 1 (before training). Significance level = 0.05. The non-significant correlations are marked with a cross.


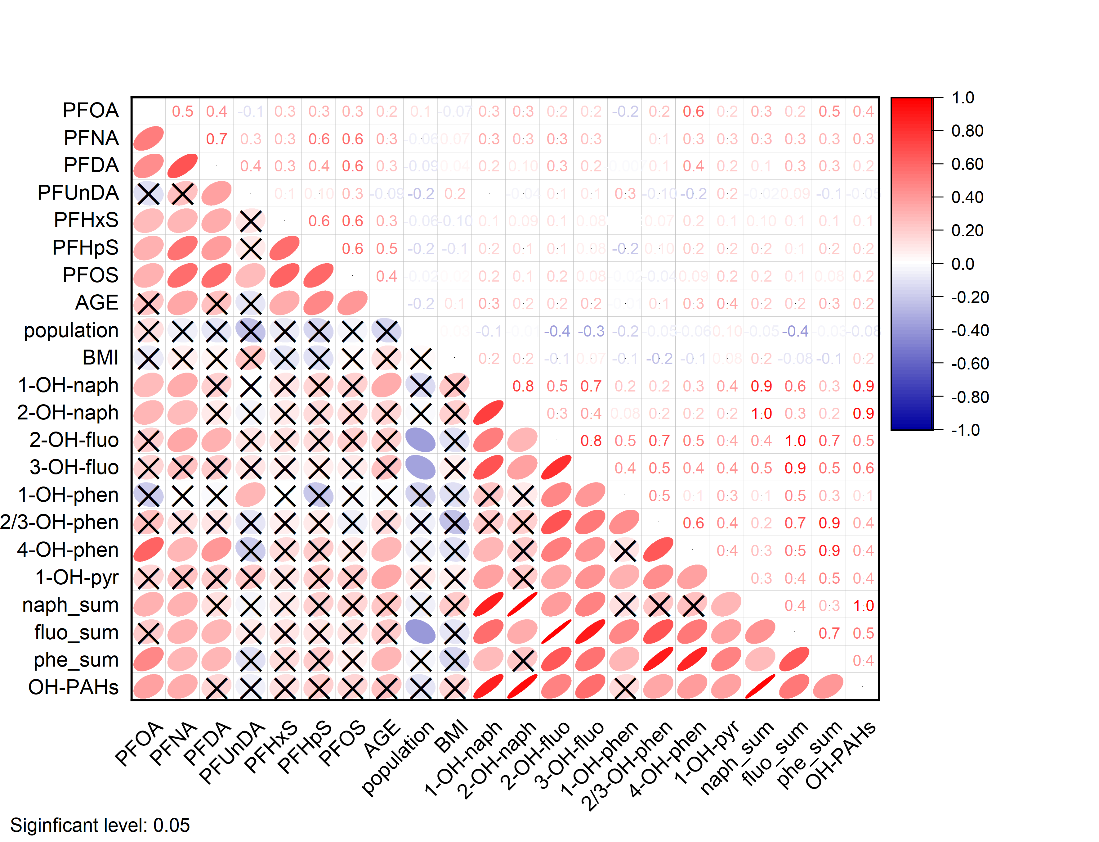


Figure SI06: Spearman’s correlation of serum PFAS concentrations, urinary OH-PAH concentrations, BMI, age, and population size in the control group in phase 1. Significance level = 0.05. The non-significant correlations are marked with a cross.

Figure SI07: Spearman’s correlation of serum PFAS concentrations, urinary OH-PAH concentrations, BMI, age, population size, and length of firefighting career in the professional firefighters and new trainees in phase 1 (before training). Significance level = 0.05. The non-significant correlations are marked with a cross.

## PFAS and OH-PAH detection frequencies and LOD

Table SI08: Detection frequencies (% of measurements above the LOD) of serum PFAS for all measurements, and each subcohort and phase. New = new trainees, prof = professionals, ctrl = control group. The number represents the phase.

PFAS, PFOA, PFNA, PFDA, PFHxS, and PFOS were the most frequently detected compounds, with a detection frequency higher than 95 %. Other frequently detected were PFUnDA (94.4 %) and PFHpS (84.5 %).

| **Subcohort**  **phase** | **Compound** | **PFPA** | **PFHxA** | **PFHpA** | **PFOA** | **PFNA** | **PFDA** | **PFUnDA** | **PFDoDA** | **PFBS** | **PFHxS** | **PFHpS** | **PFOS** |
| --- | --- | --- | --- | --- | --- | --- | --- | --- | --- | --- | --- | --- | --- |
| **All**  **(N = 393)** | DF (%) | 40.5 | 60.1 | 28.0 | 99.5 | 99.7 | 99.5 | 94.4 | 10.7 | 51.4 | 99.5 | 84.5 | 99.5 |
| **new1**  **(N = 58)** |  | 3.4 | 50.0 | 34.5 | 100.0 | 100.0 | 100.0 | 93.1 | 19.0 | 60.3 | 100.0 | 96.6 | 100.0 |
| **new2**  **(N = 58)** |  | 24.1 | 37.9 | 39.7 | 100.0 | 100.0 | 100.0 | 86.2 | 17.2 | 58.6 | 100.0 | 94.8 | 100.0 |
| **new3**  **(N = 58)** |  | 24.1 | 41.4 | 34.5 | 100.0 | 98.3 | 98.3 | 91.4 | 15.5 | 58.6 | 98.3 | 93.1 | 98.3 |
| **new4**  **(N = 58)** |  | 51.7 | 60.3 | 32.8 | 100.0 | 100.0 | 100.0 | 100.0 | 8.6 | 41.4 | 100.0 | 91.4 | 100.0 |
| **prof1**  **( N= 52)** |  | 15.4 | 53.8 | 21.2 | 100.0 | 100.0 | 100.0 | 92.3 | 1.9 | 75.0 | 100.0 | 96.2 | 100.0 |
| **ctrl1**  **(N = 55)** |  | 83.6 | 94.5 | 23.6 | 98.2 | 100.0 | 100.0 | 100.0 | 5.5 | 43.6 | 100.0 | 63.6 | 100.0 |
| **ctrl4**  **(N = 54)** |  | 83.3 | 85.2 | 7.4 | 98.1 | 100.0 | 98.1 | 98.1 | 5.6 | 22.2 | 98.1 | 53.7 | 98.1 |
|  |  |  |  |  |  |  |  |  |  |  |  |  |  |
|  | LOD (ng/ml) | 0 | 0.04 | 0.03 | 0.07 | 0.004 | 0.004 | 0.012 | 0.016 | 0.04 | 0.004 | 0.005 | 0.03 |

Table SI09: Detection frequencies (% of measurements above the LOD) of urine OH-PAH for all measurements, and each subcohort and phase. New = new trainees, prof = professionals, ctrl = control group. The number represents the phase.

1‑OH‑Naph, 2‑OH‑Naph, 2‑OH‑Fluo, and 2/3‑OH‑Phen were detected in all samples, 1‑OH‑Phen and 4‑OH‑Phen, with high frequency (88.3 % and 92.6 %, respectively) and 9‑OH‑Phen with lower frequency 47.3 %., 3‑OH‑BaP was only detected in 1.3 % of samples.

| **Subcohort**  **phase** | **Compound** | **1-OH-Naph** | **2-OH-Naph** | **2_OH-Fluo** | **3-OH-Fluo** | **1-OH-Phen** | **4-OH-Phen** | **9-OH-Phen** | **1-OH-Pyr** | **3-OH-BaP** | **2/3-OH-Phen** |
| --- | --- | --- | --- | --- | --- | --- | --- | --- | --- | --- | --- |
| **All** | DF(%) | 100.0 | 100.0 | 100.0 | 98.0 | 88.3 | 92.6 | 47.3 | 99.5 | 1.3 | 100.0 |
| **(N=393)** |  |  |  |  |  |  |  |  |  |  |  |
| **new1** |  | 100.0 | 100.0 | 100.0 | 100.0 | 81.0 | 100.0 | 72.4 | 98.3 | 5.2 | 100.0 |
| **(N=58)** |  |  |  |  |  |  |  |  |  |  |  |
| **new2** |  | 100.0 | 100.0 | 100.0 | 96.6 | 89.7 | 93.1 | 51.7 | 100.0 | 0.0 | 100.0 |
| **(N=58)** |  |  |  |  |  |  |  |  |  |  |  |
| **new3** |  | 100.0 | 100.0 | 100.0 | 100.0 | 100.0 | 100.0 | 77.6 | 100.0 | 1.7 | 100.0 |
| **(N=58)** |  |  |  |  |  |  |  |  |  |  |  |
| **new4** |  | 100.0 | 100.0 | 100.0 | 100.0 | 96.6 | 91.4 | 50.0 | 100.0 | 0.0 | 100.0 |
| **(N=58)** |  |  |  |  |  |  |  |  |  |  |  |
| **prof1 (N=52)** |  | 100.0 | 100.0 | 100.0 | 94.2 | 61.5 | 98.1 | 46.2 | 100.0 | 1.9 | 100.0 |
| **ctrl1** |  | 100.0 | 100.0 | 100.0 | 96.4 | 94.6 | 80.0 | 18.2 | 100.0 | 0.0 | 100.0 |
| **(N=55)** |  |  |  |  |  |  |  |  |  |  |  |
| **ctrl4** |  | 100.0 | 100.0 | 100.0 | 98.2 | 92.6 | 85.2 | 11.1 | 98.2 | 0.0 | 100.0 |
| **(N=54)** |  |  |  |  |  |  |  |  |  |  |  |
| **All** | LOD(ng/ml) | 0.006 | 0.006 | 0.006 | 0.006 | 0.006 | 0.006 | 0.06 | 0.006 | 0.06 | 0.006 |

## Serum PFAS levels


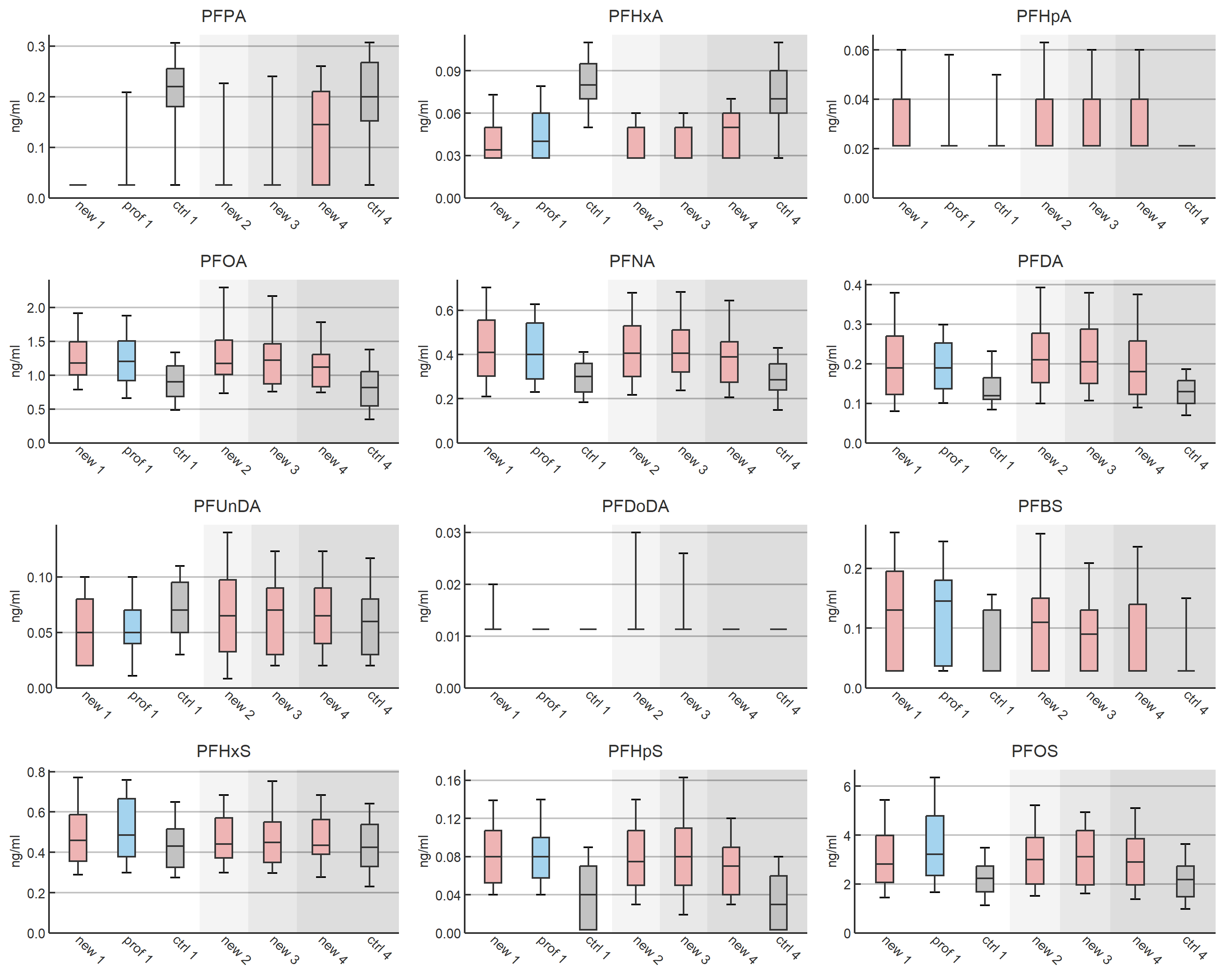
Figure SI10: Box and whiskers plot of individual serum PFAS levels in ng/ml in each subcohort and phase. The box and whiskers plot shows a median value with the 25^th^ and 75^th^ (box), and 10^th^ and 90^th^ percentiles (whiskers). Note that the concentration range on the Y-axis differs among plots. New = new trainees, prof = professionals, ctrl = control group, , the number represents the phase. Different shades of grey indicate the proceeding study phases.

TableSI11: The summary statistics for the serum PFAS levels (ng/ml) for each subcohort and phase. New = new trainees, prof = professionals, ctrl = control group. The number represents the phase.

| **Subcohort  phase** | **Compound (ng/ml)** | **PFPA** | **PFHxA** | **PFHpA** | **PFOA** | **PFNA** | **PFDA** | **PFUnDA** | **PFDoDA** | **PFBS** | **PFHxS** | **PFHpS** | **PFOS** | **∑PFAS** |
| --- | --- | --- | --- | --- | --- | --- | --- | --- | --- | --- | --- | --- | --- | --- |
| **new1**  **(N = 58)** | Median  10^th^ - 90^th^ perc.  Min - Max | 0.03  0.03 - 0.03  0.03 - 0.21 | 0.03  0.03 - 0.08  0.03 - 0.13 | 0.02  0.02 - 0.06  0.02 - 0.16 | 1.18  0.74 - 1.92  0.58 - 4.57 | 0.41  0.21 - 0.71  0.12 - 8.16 | 0.19  0.08 - 0.4  0.04 - 0.93 | 0.05  0.02 - 0.1  0.01 - 0.29 | 0.01  0.01 - 0.02  0.01 - 0.13 | 0.13  0.03 - 0.26  0.03 - 0.44 | 0.46  0.29 - 0.82  0.23 - 1.81 | 0.08  0.04 - 0.16  0 - 0.35 | 2.82  1.45 - 5.55  0.48 - 26.5 | 5.58  3.42 - 10.16  2.13 - 42.11 |
| **new2**  **(N = 58)** | Median  10th - 90th perc.  Min - Max | 0.03  0.03 - 0.24  0.03 - 0.27 | 0.03  0.03 - 0.06  0.03 - 0.07 | 0.02  0.02 - 0.07  0.02 - 0.22 | 1.18  0.71 - 2.4  0.54 - 3.69 | 0.41  0.21 - 0.72  0.1 - 5.85 | 0.21  0.1 - 0.4  0.07 - 0.9 | 0.07  0.01 - 0.14  0.01 - 0.34 | 0.01  0.01 - 0.03  0.01 - 0.15 | 0.11  0.03 - 0.3  0.03 - 0.79 | 0.44  0.3 - 0.83  0.23 - 1.54 | 0.08  0.03 - 0.14  0 - 0.35 | 3  1.41 - 5.24  0.37 - 22.9 | 5.85  3.55 - 9.77  1.64 - 34.95 |
| **new3**  **(N = 58)** | Median  10^th^ - 90^th^ perc.  Min - Max | 0.03  0.03 - 0.24  0.03 - 0.31 | 0.03  0.03 - 0.06  0.03 - 0.08 | 0.02  0.02 - 0.06  0.02 - 0.19 | 1.22  0.75 - 2.43  0.48 - 3.41 | 0.41  0.23 - 0.69  0 - 5.76 | 0.21  0.1 - 0.38  0 - 0.86 | 0.07  0.02 - 0.13  0.01 - 0.31 | 0.01  0.01 - 0.04  0.01 - 0.2 | 0.09  0.03 - 0.23  0.03 - 0.63 | 0.45  0.29 - 0.76  0 - 1.6 | 0.08  0.02 - 0.17  0 - 0.95 | 3.13  1.53 - 5.4  0.02 - 21.9 | 5.93  3.58 - 8.87  1.07 - 34.17 |
| **new4**  **(N = 58)** | Median  10^th^ - 90^th^ perc.  Min - Max | 0.15  0.03 - 0.26  0.03 - 0.39 | 0.05  0.03 - 0.07  0.03 - 0.12 | 0.02  0.02 - 0.06  0.02 - 0.14 | 1.12  0.74 - 1.93  0.36 - 3.13 | 0.39  0.2 - 0.65  0.11 - 4.82 | 0.18  0.09 - 0.41  0.06 - 0.63 | 0.07  0.02 - 0.13  0.01 - 0.3 | 0.01  0.01 - 0.01  0.01 - 0.16 | 0.03  0.03 - 0.25  0.03 - 0.33 | 0.44  0.27 - 0.69  0.22 - 1.3 | 0.07  0.03 - 0.12  0 - 0.27 | 2.9  1.38 - 5.45  0.51 - 20.7 | 5.34  3.44 - 9.59  2.05 - 31.06 |
| **prof1**  **(N = 52)** | Median  10^th^ - 90^th^ perc.  Min - Max | 0.03  0.03 - 0.21  0.03 - 0.4 | 0.04  0.03 - 0.08  0.03 - 0.18 | 0.02  0.02 - 0.06  0.02 - 0.09 | 1.21  0.66 - 1.88  0.13 - 2.53 | 0.4  0.23 - 0.63  0.06 - 0.99 | 0.19  0.1 - 0.3  0.04 - 0.58 | 0.05  0.01 - 0.1  0.01 - 0.14 | 0.01  0.01 - 0.01  0.01 - 0.05 | 0.15  0.03 - 0.25  0.03 - 0.49 | 0.49  0.3 - 0.76  0.14 - 3.67 | 0.08  0.04 - 0.14  0 - 0.47 | 3.22  1.64 - 6.37  0.43 - 17 | 6.02  4.03 - 10.17  1.31 - 20.72 |
| **ctrl1**  **(N = 55)** | Median  10^th^ - 90^th^ perc.  Min - Max | 0.22  0.03 - 0.31  0.03 - 0.37 | 0.08  0.05 - 0.11  0.03 - 0.19 | 0.02  0.02 - 0.05  0.02 - 0.23 | 0.9  0.45 - 1.35  0.05 - 1.89 | 0.3  0.18 - 0.42  0.09 - 0.95 | 0.12  0.08 - 0.24  0.04 - 0.38 | 0.07  0.03 - 0.11  0 - 0.3 | 0.01  0.01 - 0.01  0.01 - 0.08 | 0.03  0.03 - 0.16  0.03 - 0.37 | 0.43  0.27 - 0.65  0.09 - 1 | 0.04  0 - 0.09  0 - 0.13 | 2.24  1.08 - 3.62  0.53 - 7.17 | 4.65  2.8 - 6.2  1.39 - 10.74 |
| **ctrl4**  **(N = 54)** | Median  10^th^ - 90^th^ perc.  Min - Max | 0.2  0.03 - 0.31  0.03 - 0.51 | 0.07  0.03 - 0.11  0.03 - 0.23 | 0.02  0.02 - 0.02  0.02 - 0.25 | 0.82  0.34 - 1.38  0.05 - 2.27 | 0.29  0.14 - 0.45  0.07 - 0.86 | 0.13  0.07 - 0.19  0 - 0.43 | 0.06  0.02 - 0.12  0 - 0.24 | 0.01  0.01 - 0.01  0.01 - 0.04 | 0.03  0.03 - 0.15  0.03 - 0.66 | 0.43  0.23 - 0.65  0 - 1.01 | 0.03  0 - 0.08  0 - 0.15 | 2.19  0.97 - 3.78  0.02 - 6.95 | 4.37  2.33 - 6.19  1.14 - 10.2 |
|  |  |  |  |  |  |  |  |  |  |  |  |  |  |  |

## Urinary OH-PAHs levels

Table SI12: Descriptive statistics for non-adjusted urine OH-PAH levels (ng/ml) in each subcohort and phase. New = new trainees, prof = professionals, ctrl = control group. The number represents the phase.

| **Subcohort  phase** | **Compound (ng/ml)** | **1-OH-Naph** | **2-OH-Naph** | **2-OH-Fluo** | **3-OH-Fluo** | **1-OH-Phen** | **4-OH-Phen** | **9-OH-Phen** | **1-OH-Pyr** | **3-OH-BaP** | **2/3-OH-Phen** |
| --- | --- | --- | --- | --- | --- | --- | --- | --- | --- | --- | --- |
| **new1**  **(N = 58)** | Median  10^th^ - 90^th^ perc.  Min - Max | 3.32  0.85 - 12.6  0.51 - 21.6 | 6.41  2.38 - 16.9  0.39 - 35.4 | 0.44  0.19 - 0.95  0.06 - 2.18 | 0.13  0.04 - 0.33  0.02 - 0.63 | 0.04  0 - 0.11  0 - 0.25 | 0.61  0.22 - 1.24  0.03 - 3.23 | 0.31  0.04 - 0.82  0.04 - 1.61 | 0.15  0.06 - 0.36  0 - 0.46 | 0.04  0.04 - 0.04  0.04 - 0.45 | 0.27  0.11 - 0.86  0.05 - 1.61 |
| **new2**  **(N = 58)** | Median  10^th^ - 90^th^ perc.  Min - Max | 5.96  2.05 - 28.5  0.62 - 86.4 | 11.5  3.64 - 38.6  1.33 - 91.2 | 0.43  0.15 - 1.8  0.07 - 6.02 | 0.1  0.03 - 0.33  0 - 1.08 | 0.09  0 - 0.28  0 - 0.84 | 0.39  0.03 - 2.05  0 - 6.27 | 0.21  0.04 - 1.14  0.04 - 3.93 | 0.12  0.03 - 0.36  0.02 - 1.1 | 0.04  0.04 - 0.04  0.04 - 0.04 | 0.23  0.06 - 0.58  0.02 - 1.99 |
| **new3**  **(N = 58)** | Median  10^th^ - 90^th^ perc.  Min - Max | 10.9  2.29 - 33.4  1.2 - 72.6 | 15.05  2.88 - 45.6  1.45 - 82.8 | 0.8  0.23 - 2.58  0.09 - 3.9 | 0.15  0.04 - 0.52  0.02 - 0.66 | 0.2  0.05 - 0.73  0.02 - 1.99 | 0.93  0.09 - 4.47  0.02 - 10.8 | 0.58  0.04 - 2.35  0.04 - 4.73 | 0.32  0.09 - 1.1  0.05 - 2.16 | 0.04  0.04 - 0.04  0.04 - 0.24 | 0.46  0.16 - 1.98  0.07 - 3.03 |
| **new4**  **(N = 58)** | Median  10^th^ - 90^th^ perc.  Min - Max | 2.32  0.77 - 8.55  0.54 - 36.9 | 6.39  2.26 - 16.1  1.37 - 34.9 | 0.41  0.21 - 1.04  0.12 - 3.31 | 0.09  0.03 - 0.37  0.02 - 1.43 | 0.13  0.03 - 0.38  0 - 3.02 | 0.25  0.02 - 1.38  0 - 5.07 | 0.15  0.04 - 0.65  0.04 - 1.27 | 0.22  0.09 - 0.69  0.05 - 2.08 | 0.04  0.04 - 0.04  0.04 - 0.04 | 0.29  0.16 - 0.88  0.07 - 3.53 |
| **prof1**  **(N = 52)** | Median  10^th^ - 90^th^ perc.  Min - Max | 2.1  0.85 - 5.43  0.15 - 12.9 | 5.6  2.06 - 13.9  1.26 - 31 | 0.34  0.17 - 0.71  0.1 - 1.8 | 0.09  0.03 - 0.23  0 - 0.35 | 0.03  0 - 0.18  0 - 0.86 | 0.46  0.09 - 0.86  0 - 1.19 | 0.04  0.04 - 0.53  0.04 - 1.29 | 0.11  0.05 - 0.21  0.03 - 0.55 | 0.04  0.04 - 0.04  0.04 - 0.28 | 0.21  0.08 - 0.44  0.03 - 0.84 |
| **ctrl1**  **(N = 55)** | Median  10^th^ - 90^th^ perc.  Min - Max | 1.69  0.73 - 4.46  0.4 - 12.6 | 5.21  2.12 - 12.9  1.62 - 74.1 | 0.36  0.15 - 0.78  0.07 - 1.33 | 0.07  0.02 - 0.18  0 - 0.26 | 0.17  0.05 - 0.46  0 - 1.96 | 0.05  0 - 0.57  0 - 3.21 | 0.04  0.04 - 0.46  0.04 - 1.12 | 0.1  0.03 - 0.22  0.02 - 0.57 | 0.04  0.04 - 0.04  0.04 - 0.04 | 0.25  0.11 - 0.68  0.05 - 1.74 |
| **ctrl4**  **(N = 54)** | Median  10^th^ - 90^th^ perc.  Min - Max | 2.1  0.74 - 8.41  0.28 - 28.1 | 4.44  1.66 - 30.4  1.27 - 58.6 | 0.36  0.15 - 1.21  0.06 - 3.36 | 0.09  0.03 - 0.35  0 - 0.94 | 0.22  0.04 - 0.64  0 - 2.86 | 0.05  0 - 0.76  0 - 1.54 | 0.04  0.04 - 0.32  0.04 - 0.71 | 0.12  0.03 - 0.28  0 - 2.06 | 0.04  0.04 - 0.04  0.04 - 0.04 | 0.3  0.1 - 0.74  0.07 - 3.8 |
|  |  |  |  |  |  |  |  |  |  |  |  |

Table SI13: Descriptive statistics for specific gravity-adjusted urine OH-PAH levels (ng/ml) in each subcohort and phase. New = new trainees, prof = professionals, ctrl = control group. The number represents the phase.

| **Subcohort phase** | **Compound (ng/ml)** | **1-OH-Naph** | **2-OH-Naph** | **2-OH-Fluo** | **3-OH-Fluo** | **1-OH-Phen** | **4-OH-Phen** | **9-OH-Phen** | **1-OH-Pyr** | **3-OH-BaP** | **2/3-OH-Phen** |
| --- | --- | --- | --- | --- | --- | --- | --- | --- | --- | --- | --- |
| **new1**  **(N = 58)** | Median  10^th^ - 90^th^ perc.  Min - Max | 2.94  0.98 - 8.87  0.62 - 17.85 | 5.48  2.89 - 16.92  1.91 - 26.18 | 0.36  0.24 - 0.79  0.14 - 1.6 | 0.11  0.04 - 0.34  0.02 - 0.51 | 0.04  0.01 - 0.09  0 - 0.21 | 0.57  0.29 - 1.07  0.03 - 2.25 | 0.28  0.04 - 0.65  0.03 - 1.33 | 0.15  0.06 - 0.29  0.01 - 0.38 | 0.04  0.03 - 0.06  0.03 - 2.74 | 0.25  0.13 - 0.64  0.08 - 1.4 |
| **new2**  **(N = 58)** | Median  10^th^ - 90^th^ perc.  Min - Max | 12.68  4.79 - 42.95  2.12 - 67.98 | 19.67  8.07 - 50.42  5.46 - 85.5 | 0.76  0.44 - 2.18  0.28 - 4.48 | 0.17  0.1 - 0.35  0.02 - 0.8 | 0.15  0.03 - 0.42  0.01 - 0.66 | 0.84  0.03 - 2.89  0.01 - 4.66 | 0.35  0.05 - 1.24  0.03 - 2.92 | 0.2  0.11 - 0.5  0.08 - 0.82 | 0.07  0.04 - 0.24  0.03 - 0.33 | 0.36  0.21 - 0.71  0.11 - 1.48 |
| **new3**  **(N = 58)** | Median  10^th^ - 90^th^ perc.  Min - Max | 13.3  6.44 - 35.34  4.14 - 62.96 | 18.11  8.13 - 41.39  4.31 - 53.94 | 0.99  0.61 - 2.05  0.4 - 3.39 | 0.2  0.11 - 0.41  0.06 - 0.55 | 0.25  0.12 - 0.68  0.07 - 1.3 | 1.34  0.13 - 3.4  0.06 - 7.04 | 0.87  0.06 - 1.79  0.03 - 3.08 | 0.46  0.23 - 0.86  0.14 - 1.42 | 0.05  0.03 - 0.17  0.03 - 0.75 | 0.62  0.38 - 1.51  0.25 - 2.63 |
| **new4**  **(N = 58)** | Median  10^th^ - 90^th^ perc.  Min - Max | 1.95  0.81 - 8.26  0.5 - 28.38 | 5.37  1.9 - 11.77  1.48 - 26.85 | 0.38  0.2 - 0.74  0.12 - 2.55 | 0.07  0.03 - 0.3  0.02 - 0.83 | 0.1  0.03 - 0.3  0.01 - 2.32 | 0.29  0.02 - 1.13  0 - 4.53 | 0.13  0.03 - 0.53  0.03 - 0.74 | 0.22  0.08 - 0.5  0.06 - 1.6 | 0.04  0.03 - 0.06  0.02 - 0.09 | 0.26  0.14 - 0.58  0.11 - 2.72 |
| **prof1**  **(N = 52)** | Median  10^th^ - 90^th^ perc.  Min - Max | 2.43  0.87 - 5.03  0.16 - 10.94 | 6.37  2.56 - 13.38  1.36 - 25.92 | 0.39  0.23 - 0.77  0.11 - 1.51 | 0.11  0.03 - 0.23  0 - 0.31 | 0.03  0 - 0.17  0 - 0.76 | 0.49  0.07 - 0.99  0.01 - 1.46 | 0.1  0.04 - 0.7  0.03 - 1.4 | 0.11  0.06 - 0.2  0.04 - 0.6 | 0.05  0.03 - 0.08  0.02 - 0.4 | 0.2  0.12 - 0.54  0.03 - 0.91 |
| **ctrl1**  **(N = 55)** | Median  10^th^ - 90^th^ perc.  Min - Max | 1.6  0.69 - 3.78  0.43 - 7.83 | 4.73  2.03 - 11.07  1.36 - 46.02 | 0.3  0.16 - 0.58  0.09 - 1.03 | 0.06  0.02 - 0.15  0.01 - 0.25 | 0.16  0.04 - 0.39  0 - 1.39 | 0.04  0 - 0.49  0 - 2.39 | 0.04  0.03 - 0.37  0.02 - 0.77 | 0.08  0.04 - 0.16  0.01 - 0.4 | 0.04  0.03 - 0.05  0.02 - 0.09 | 0.24  0.11 - 0.49  0.08 - 1.23 |
| **ctrl4**  **(N = 54)** | Median  10^th^ - 90^th^ perc.  Min - Max | 1.7  0.76 - 7.54  0.45 - 18.67 | 3.81  2.12 - 20.54  1.51 - 47.07 | 0.36  0.17 - 0.94  0.08 - 2.23 | 0.08  0.03 - 0.3  0.01 - 0.65 | 0.18  0.04 - 0.56  0.01 - 1.9 | 0.05  0.01 - 0.76  0 - 1.76 | 0.04  0.03 - 0.26  0.02 - 0.53 | 0.1  0.05 - 0.29  0.01 - 1.37 | 0.03  0.03 - 0.07  0.02 - 0.09 | 0.26  0.12 - 0.54  0.07 - 2.29 |
|  |  |  |  |  |  |  |  |  |  |  |  |

Table SI14: The summary statistics for the creatinine-adjusted urine OH-PAH levels (µg/g_creat_) for each subcohort and phase. New = new trainees, prof = professionals, ctrl = control group. The number represents the phase.

| **Subcohort  phase** | **Compound**  **(µg/g_creat_)** | **1-OH-Naph** | **2-OH-Naph** | **2-OH-Fluo** | **3-OH-Fluo** | **1-OH-Phen** | **4-OH-Phen** | **9-OH-Phen** | **1-OH-Pyr** | **3-OH-BaP** | **2/3-OH-Phen** | **∑Naph** | **∑Fluo** | **∑Phen** | **∑OH-PAH** |
| --- | --- | --- | --- | --- | --- | --- | --- | --- | --- | --- | --- | --- | --- | --- | --- |
| **new1**  **(N = 58)** | Median  10^th^ - 90^th^ perc.  Min - Max | 1.97  0.56 - 5.65  0.36 - 8.54 | 3.82  1.64 - 11.94  1.1 - 19.75 | 0.24  0.14 - 0.47  0.1 - 0.98 | 0.07  0.02 - 0.16  0.02 - 0.27 | 0.02  0 - 0.05  0 - 0.13 | 0.34  0.18 - 0.72  0.02 - 1.35 | 0.17  0.03 - 0.35  0.02 - 0.83 | 0.09  0.04 - 0.17  0.01 - 0.27 | 0.02  0.01 - 0.05  0.01 - 1.53 | 0.17  0.08 - 0.35  0.05 - 0.62 | 5.37  2.46 - 15.8  1.67 - 23.82 | 0.3  0.17 - 0.63  0.12 - 1.25 | 0.72  0.37 - 1.26  0.17 - 2.2 | 6.51  3.29 - 17.74  2.62 - 24.69 |
| **new2**  **(N = 58)** | Median  10^th^ - 90^th^ perc.  Min - Max | 8.44  3.15 - 24.34  1.45 - 41.76 | 12.81  6.19 - 31.09  3.74 - 70.62 | 0.49  0.28 - 1.13  0.18 - 2.09 | 0.11  0.06 - 0.2  0.02 - 0.38 | 0.09  0.02 - 0.23  0.01 - 0.29 | 0.56  0.02 - 1.61  0.01 - 2.45 | 0.2  0.04 - 0.71  0.01 - 1.36 | 0.13  0.08 - 0.27  0.07 - 0.5 | 0.05  0.02 - 0.16  0.01 - 0.21 | 0.25  0.15 - 0.43  0.09 - 0.69 | 21.44  9.3 - 59.21  5.2 - 101.39 | 0.61  0.35 - 1.32  0.22 - 2.47 | 1.09  0.5 - 2.39  0.27 - 4.52 | 23.65  10.43 - 63.77  5.84 - 105.17 |
| **new3**  **(N = 58)** | Median  10^th^ - 90^th^ perc.  Min - Max | 8.99  4.61 - 20.07  3.33 - 51.5 | 11.98  6.88 - 27.06  3.94 - 37.32 | 0.73  0.41 - 1.31  0.3 - 1.67 | 0.14  0.08 - 0.22  0.04 - 0.35 | 0.17  0.1 - 0.33  0.04 - 0.61 | 0.99  0.1 - 2.22  0.05 - 2.7 | 0.65  0.05 - 1.13  0.02 - 1.53 | 0.31  0.16 - 0.52  0.11 - 0.67 | 0.04  0.01 - 0.12  0.01 - 0.52 | 0.43  0.28 - 0.83  0.15 - 1.38 | 21.35  11.64 - 44.99  7.73 - 81 | 0.88  0.5 - 1.49  0.34 - 1.94 | 2.28  0.77 - 4.16  0.53 - 5.25 | 25.09  13.19 - 50.24  9.15 - 88.21 |
| **new4**  **(N = 58)** | Median  10^th^ - 90^th^ perc.  Min - Max | 1.17  0.41 - 4.38  0.3 - 10.57 | 3.02  1.34 - 7.77  0.74 - 13.04 | 0.21  0.13 - 0.48  0.11 - 0.95 | 0.04  0.02 - 0.19  0.01 - 0.37 | 0.06  0.01 - 0.17  0 - 0.87 | 0.17  0.01 - 0.68  0 - 3.52 | 0.07  0.02 - 0.27  0.01 - 0.58 | 0.12  0.05 - 0.29  0.04 - 0.61 | 0.02  0.01 - 0.03  0.01 - 0.05 | 0.14  0.09 - 0.32  0.06 - 1.01 | 4.75  1.83 - 10.88  1.07 - 20.57 | 0.24  0.14 - 0.71  0.12 - 1.21 | 0.53  0.2 - 1.39  0.14 - 4.88 | 5.73  2.27 - 12.8  1.49 - 24.63 |
| **prof1**  **(N = 52)** | Median  10^th^ - 90^th^ perc.  Min - Max | 1.64  0.51 - 3.65  0.13 - 6 | 4.18  1.38 - 9.69  0.96 - 14.69 | 0.26  0.14 - 0.48  0.09 - 1.1 | 0.06  0.02 - 0.16  0 - 0.3 | 0.02  0 - 0.1  0 - 0.31 | 0.32  0.04 - 0.59  0 - 0.98 | 0.07  0.02 - 0.4  0.02 - 0.88 | 0.07  0.04 - 0.14  0.03 - 0.37 | 0.03  0.02 - 0.05  0.01 - 0.2 | 0.14  0.07 - 0.3  0.03 - 0.57 | 6.11  1.87 - 13.19  1.23 - 17.55 | 0.32  0.18 - 0.63  0.09 - 1.41 | 0.59  0.32 - 1.28  0.15 - 2.14 | 7  2.57 - 15.18  1.71 - 18.45 |
| **ctrl1**  **(N = 55)** | Median  10^th^ - 90^th^ perc.  Min - Max | 0.93  0.37 - 2.32  0.28 - 3.48 | 2.76  1.22 - 5.45  0.64 - 18.03 | 0.18  0.09 - 0.32  0.07 - 0.52 | 0.03  0.01 - 0.07  0 - 0.22 | 0.09  0.02 - 0.21  0 - 0.74 | 0.02  0 - 0.39  0 - 1.39 | 0.02  0.01 - 0.2  0.01 - 0.42 | 0.04  0.02 - 0.1  0.01 - 0.21 | 0.02  0.01 - 0.04  0.01 - 0.06 | 0.12  0.06 - 0.29  0.05 - 0.65 | 3.74  1.63 - 7.01  0.99 - 21.09 | 0.21  0.1 - 0.38  0.07 - 0.74 | 0.29  0.15 - 1.1  0.09 - 1.97 | 4.44  2.24 - 8.42  1.18 - 21.76 |
| **ctrl4**  **(N = 54)** | Median  10^th^ - 90^th^ perc.  Min - Max | 0.97  0.49 - 3.97  0.22 - 6.98 | 2.33  1.15 - 8.12  0.59 - 18.43 | 0.2  0.09 - 0.41  0.05 - 0.66 | 0.04  0.02 - 0.12  0.01 - 0.32 | 0.09  0.02 - 0.38  0.01 - 0.83 | 0.02  0 - 0.48  0 - 1.71 | 0.02  0.01 - 0.15  0.01 - 0.34 | 0.05  0.02 - 0.14  0.01 - 0.4 | 0.02  0.01 - 0.05  0.01 - 0.08 | 0.15  0.07 - 0.36  0.04 - 1.29 | 3.42  1.81 - 13.05  0.8 - 23.71 | 0.25  0.11 - 0.58  0.06 - 0.97 | 0.34  0.15 - 1.37  0.09 - 2.06 | 4.42  2.4 - 14.34  0.98 - 24.58 |
|  |  |  |  |  |  |  |  |  |  |  |  |  |  |  |  |


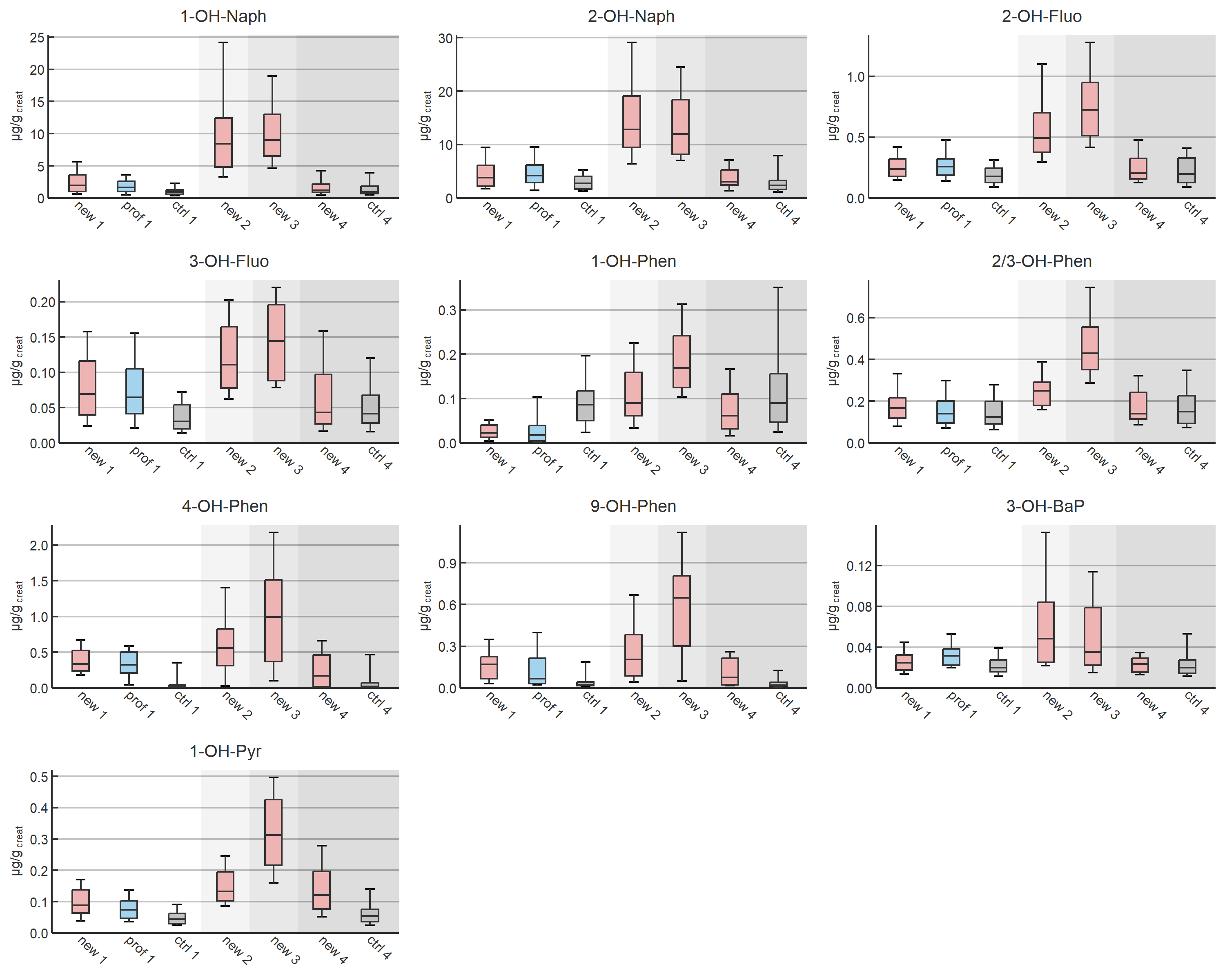
Figure SI15: Box and whiskers plots of urine creatinine-adjusted individual OH-PAH levels in µg/g_creat_ in each subcohort and phase. The box and whiskers plot shows a median value with 25^th^,and 75^th^ (box) and 10^th^ and 90^th^ percentiles (whiskers). Note that the concentration range on the Y-axis differs among plots. New = new trainees, prof = professionals, ctrl = control group, the number represents the phase. Different shades of grey indicate the proceeding study phases.


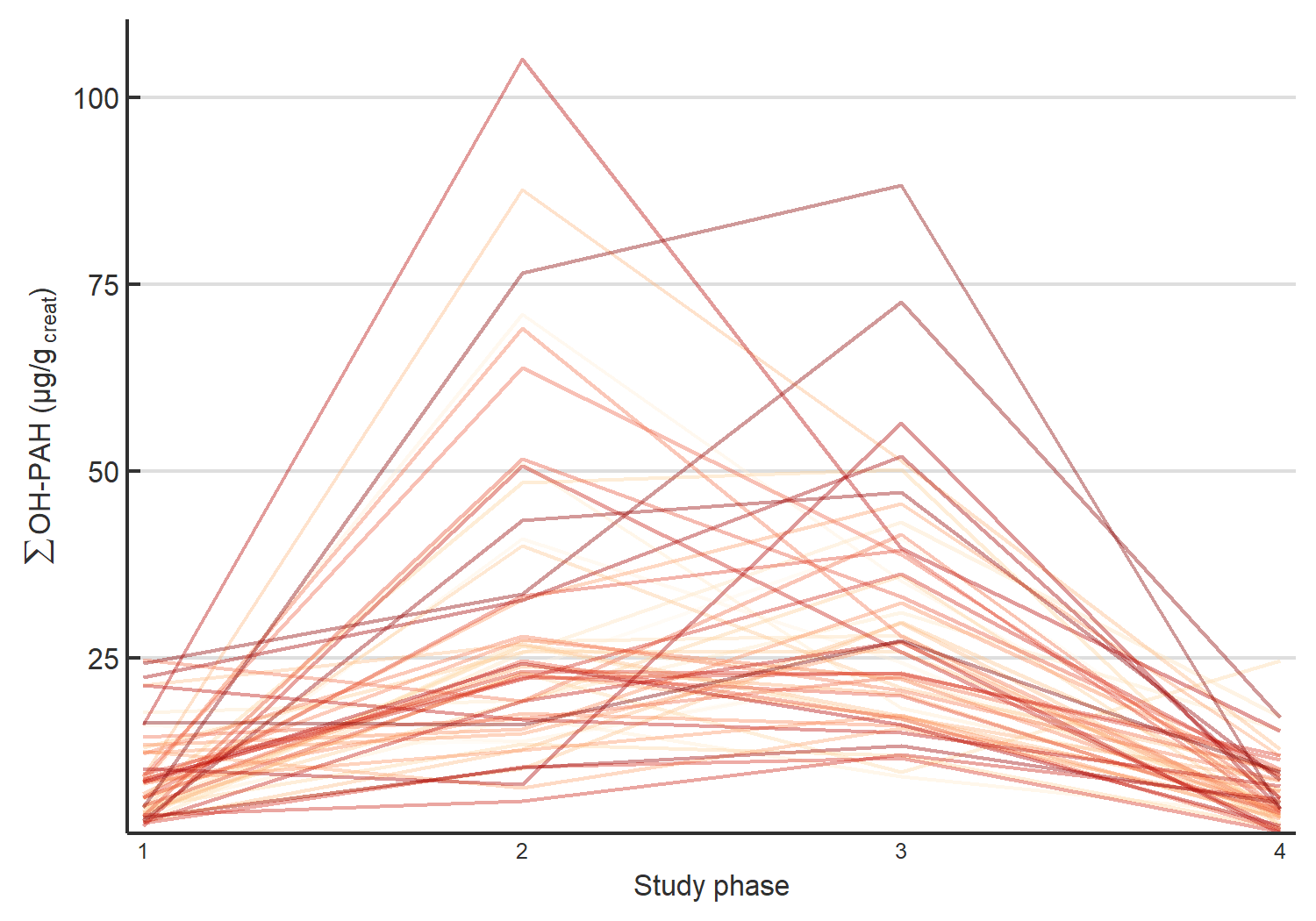


Figure SI16: Course of ΣPAH urine levels (µg/g_creat_) in individual participants within the study phases. There was no statistically significant difference in the PAH levels between phase 2 and phase 3 of the study. However, the line plot shows that for some participants, there is an increase of PAH levels in phase 2 followed by a decrease in phase 3, while for others, the peak PAH levels occur in phase 3.

## Population characteristics and questionnaire data

Table SI17: Results of questionnaires and population in the place of residence of the participants. New = new trainees, prof = professionals, ctrl = control group.

|  |  | **new** | **prof** | **ctrl** |
| --- | --- | --- | --- | --- |
| **Participants that filled out questionnaires** |  | 59 | 52 | 55 |
| **Age (years)** | Median  10th - 90th perc.  Min. - Max. | 24.5  21 - 31  19 - 34 | 28.0  23 - 33  20 - 35 | 26.0  20 - 32  18 - 35 |
| **BMI (kg/m^2^)** | Median  10th - 90th perc.  Min. - Max. | 26.3  22.6 - 30.3  20.7 - 33.4 | 26.2  22.9 - 29  21.1 - 32.2 | 24.6  21.6 - 28.6  18.4 - 30.9 |
| **Population size in place of residence (thousands of inhabitants)** | Median  10th - 90th perc.  Min. - Max. | 3.4  0.3 – 48.8  0.1 –1 308.6 | 3.4  0.5 – 172.4  0.1– 1 308.6 | 75  1.7 – 380.7  0.4 – 380.7 |
| **Infectious or chronic disease (%)** | Yes  No | 0.0  100.0 | 0.0  100.0 | 5.5  94.6 |
| **Health (subjective assessment, %)** | Always healthy and well  Mostly healthy and well  Often do not feel well | 50.9  49.2  0.0 | 67.3  32.7  0.0 | 56.4  41.8  1.8 |
| **Job (%)** | Firefighter  Student  IT  Office  Other | 100.0  0.0  0.0  0.0  0.0 | 100.0  0.0  0.0  0.0  0.0 | 0.0  45.5  14.6  12.7  27.2 |
| **Length of firefighting career (years)** | Median  10th - 90th perc.  Min. - Max. | 0.5  0.25 - 1  0 - 5 | 3.3  1 - 10  0.5 - 14 | 0.0  0.0  0.0 |
| **Smoking (%)** | Yes  No | 0.0  100.0 | 0.0  100.0 | 0.0  100.0 |
| **Former smoking (%)** | Yes  No | 11.9  88.1 | 19.2  80.8 | 7.3  92.7 |
| **If a former smoker (years since quitting)** | Median  10th - 90th perc.  Min. - Max | 4.0  0.5 – 10  0.5 - 10 | 0.5  0.2 - 5  0.1 = 6 | 3.5  0.6 – 8  0.6 - 8 |
| **Contact with a large fire in the last 6 months (%)** | Two or more times  One time  Never | 22.0  32.2  45.8 | 59.6  21.2  19.2 | 5.5  0.0  90.9 |
| **Central heating at home (%)** | Yes  No | 25.4  74.6 | 28.9  71.2 | 43.6  56.4 |
| **Heating fuel (%)** | Gas  Electric  Coal  Biomass | 48.3  6.9  12.1  29.3 | 40.4  11.5  17.3  25.0 | 45.5  14.6  1.8  10.9 |
| **Fireplace at home (%)** | Yes  No | 25.5  74.5 | 17.1  82.9 | 22.9  77.1 |
| **Contact with firefighting foams in the last year (%)** | Two or more times  One time  Never | 0.0  25.4  69.5 | 34.6  40.4  25.0 | 0.0  1.8  98.2 |
| **Work in ski service (%)** | Yes  No | 0.0  100.0 | 1.9  98.1 | 0.0  100.0 |
| **Skiing (%)** | Yes  No | 74.6  25.4 | 80.8  19.2 | 74.6  25.5 |
| **Ski self-service (% of those who ski)** | Yes  No | 15.9  84.1 | 16.7  83.3 | 12.2  87.8 |
| **Diet (%)** | Mixed diet  Vegetarian  Vegan | 100.0  0.0  0.0 | 100.0  0.0  0.0 | 100.0  0.0  0.0 |
| **Use of food supplements (%)** | Yes  No | 27.1  72.9 | 17.3  82.7 | 56.4  43.6 |
| **Water source at home (%)** | Pipeline  Well  Bottled  Mixed sources | 71.2  18.6  3.4  6.8 | 82.7  9.6  3.9  3.9 | 92.7  1.8  1.8  3.6 |
| **Use of water filter at home (%)** | Yes  No  Do not know | 4.4  80.4  15.2 | 17.8  75.6  6.7 | 13.2  71.7  15.1 |
| **Water source at work (%)** | Pipeline  Bottled  Mixed sources | 83.1  11.9  5.1 | 90.4  5.8  3.9 | 85.5  12.7  1.8 |
| **Use of water filter at work (%)** | Yes  No  Do not know | 11.5  34.6  53.9 | 22.5  57.1  20.4 | 16.7  56.3  27.1 |
| **Use of dental floss (%)** | Yes  No | 22.0  77.9 | 23.1  76.9 | 41.8  58.2 |
| **Blood donation in the last 12 months (%)** | Yes  No | 20.3  79.7 | 28.9  71.2 | 18.2  81.8 |
| **Frequency of using GoreTex jacket (%)** | Often  Sometimes  Exceptionally  Never  Do not know | 11.9  30.5  25.4  20.3  11.9 | 11.5  51.9  19.2  13.5  3.9 | 1.8  21.8  27.3  34.6  14.6 |
| **Frequency of using GoreTex trousers (?)** | Often  Sometimes  Exceptionally  Never  Do not know | 8.5  11.9  33.9  35.6  10.2 | 7.7  30.8  28.9  28.9  3.9 | 0.0  10.9  21.8  54.6  12.7 |
| **Frequency of using GoreTex gloves (%)** | Often  Sometimes  Exceptionally  Never  Do not know | 10.2  8.5  27.1  42.4  11.9 | 5.8  19.2  38.5  26.9  9.6 | 0.0  9.1  18.2  61.8  10.9 |
| **Frequency of using GoreTex boots (%)** | Often  Sometimes  Exceptionally  Never  Do not know | 8.5  30.5  20.3  30.5  10.2 | 19.2  38.6  26.9  9.6  5.8 | 5.5  18.2  23.6  40.0  12.7 |
| **Frequency of eating grilled food (%)** | Daily and more times per week  Once per week to once per month  Once per month and less  Never | 11.9  71.2  17.0  0.0 | 19.2  57.7  23.1  0.0 | 16.4  61.8  20.0  1.8 |
| **Frequency of eating fried food (%)** | Daily and more times per week  Once per week to once per month  Once per month and less  Never | 20.3  62.7  17.0  0.0 | 19.2  61.5  19.2  0.0 | 27.3  54.6  16.4  1.8 |
| **Frequency of eating fresh and frozen fish (%)** | Daily and more times per week  Once per week to once per month  Once per month and less  Never | 6.8  44.1  42.4  6.8 | 5.8  46.2  36.5  11.5 | 12.7  40.0  41.8  5.5 |
| **Frequency of eating smoked fish (%)** | Daily and more times per week  Once per week to once per month  Once per month and less  Never | 0.0  8.5  61.0  30.5 | 0.0  11.5  50.0  38.5 | 1.8  3.6  69.1  25.5 |
| **Frequency of eating fish products (%)** | Daily and more times per week  Once per week to once per month  Once per month and less  Never | 6.8  45.8  28.8  18.6 | 3.9  28.9  48.1  19.2 | 10.9  45.5  34.6  9.1 |
| **Frequency of eating eggs and eggs products (%)** | Daily and more times per week  Once per week to once per month  Once per month and less  Never | 55.9  35.6  8.5  0.0 | 61.5  34.6  3.9  0.0 | 81.8  14.6  3.6  0.0 |
| **Frequency of eating microwave popcorn (%)** | Daily and more times per week  Once per week to once per month  Once per month and less  Never | 1.7  8.5  42.4  47.5 | 1.9  1.9  38.5  57.7 | 0.0  10.9  34.6  54.6 |
| **Frequency of eating smoked products (%)** | Daily and more times per week  Once per week to once per month  Once per month and less  Never | 42.4  42.4  15.3  0.0 | 53.9  38.5  7.7  0.0 | 52.7  32.7  10.9  3.6 |
| **Frequency of eating fries and baked potatoes (%)** | Daily and more times per week  Once per week to once per month  Once per month and less  Never | 10.2  54.2  30.5  5.1 | 3.9  59.6  34.6  1.9 | 10.9  60.0  25.5  3.6 |
| **Frequency of eating chips (%)** | Daily and more times per week  Once per week to once per month  Once per month and less  Never | 3.4  40.7  50.9  5.1 | 5.8  26.9  57.7  9.6 | 3.6  34.6  54.6  7.3 |
| **Frequency of eating fast food (%)** | Daily and more times per week  Once per week to once per month  Once per month and less  Never | 3.4  45.8  47.5  3.4 | 0.0  42.3  50.0  7.7 | 9.1  29.1  56.4  5.5 |

Professional firefighters are slightly, but significantly (p < 0.05) older than the new trainees. It corresponds with their firefighting career length (professionals are older and have been working longer as firefighters than the new trainees). Also, BMI in firefighters (new trainees and professionals) is significantly higher than in the control group (p < 0.05). The controls reside in areas with a higher median population size than the new trainees and the professionals.

The professionals have the highest percentage of former smokers and at the same time lower median of years since quitting smoking, than the control group and the new trainees. The professionals have been more frequently exposed to large fires in the past 6 months, and firefighting foams in the last year, than the new trainees before the training and the control group. More controls use central heating than the new trainees and the professionals. Natural gas and biomass prevail as sources of heating for professionals and new trainees, while it is natural gas and electric heating for controls.

The percentage of participants who are skiing is very similar in new trainees, and the controls, and slightly higher in professionals. Approximately the same percentage of skiing participants are preparing the ski surface themselves in all three groups. In general, professionals seem to wear GoreTex clothes more frequently than the controls and new trainees.

All participants are on a mixed diet. The controls showed the highest percentage of participants using dietary supplements from all three groups. Among supplements, vitamins, proteins, and immune modifiers prevailed in professionals and controls, while proteins, Branched Chain Amino Acids (BCAA), and vitamins were in the new trainees.

Water pipeline and water well is the main water source at home for new trainees and professionals, while most of the controls use water pipeline. At work, the water pipeline prevails as the main water source in all groups, but some of the participants also drink bottled water. Most of the participants do not use a water filter at home, while at work, participants do not use a filter or do not know.

Professionals are the most frequent blood donors of the three groups. The controls use dental floss with a higher frequency than the two other groups.

## Lifestyle factors and the exposure

The controls reside in areas with a higher median population size than the new trainees and the professionals. A weak but significant negative correlation (r = 0.27-0.38) between population and ΣFlu levels was observed in new trainees and the control group, and between population and FUnDA and PFHxS (r = 0.3-0.39) in new trainees. The effect of different heating fuels in the household on the OH-PAH levels was not observed.

Neither the use of dental floss or food supplements nor skiing did significantly contribute to PFAS exposure, and no increase in OH-PAH levels was observed in former smokers.

All participants were on a mixed diet. No effect of the consumption of grilled, fried, smoked, baked food, or fast food on the OH-PAH levels was observed. Consumption of fish products did not significantly contribute to PFAS exposure.

No statistically significant differences in PFAS concentrations were found in participants who used/not used firefighting foams or GoreTex clothes in the last year. In general, according to the information in the questionnaire, professionals wear GoreTex clothes and use firefighting foams more frequently than the controls and new trainees. It should be noted that the questionnaire was soliciting information specifically on the use of GoreTex clothes in participants’ free time, not during occupational activities.

The professionals have been more frequently exposed to large fires in the past 6 months, and firefighting foams in the last year, than the new trainees before the training and the control group. But there was no statistically significant difference in the OH-PAH or PFAS levels in participants who were in contact with a large fire in the last 6 months and/or firefighting foams in the last year.

The statistical significance of differences in concentrations of PFAS and OH-PAH may be affected by lower sample sizes in some subsets created by occupational and lifestyle factors. While for OH‑PAH participants were evaluated together in the first phase (N = 166), for PFAS, subcohorts of participants were assessed separately (new = 59, ctrl = 55, prof = 52), due to statistically different concentrations between subcohorts. The minimum number of samples for testing the occupational and lifestyle factor categories was 25 urine samples for OH-PAH (also accounting for urinary contaminant variability) and 10 blood samples for PFAS. Categories with fewer samples (e.g., fish consumption, and use of Goretex clothing) were combined into fewer categories or converted to Y/N responses.

| 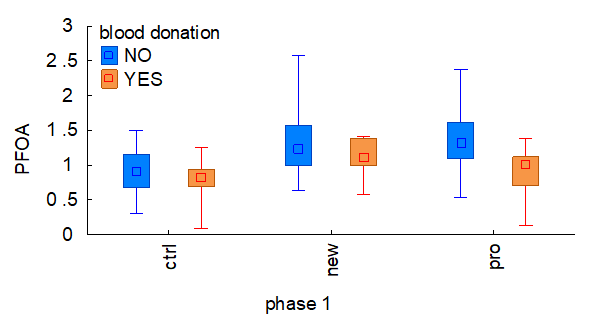 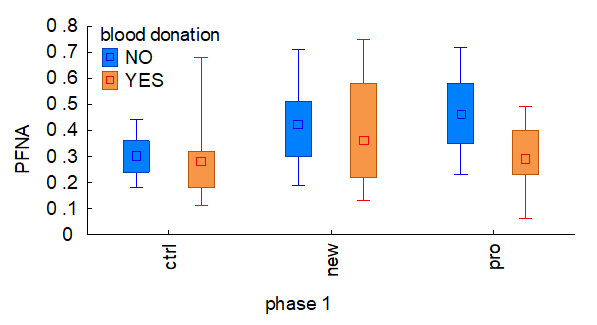 |
| --- |
|  |
| 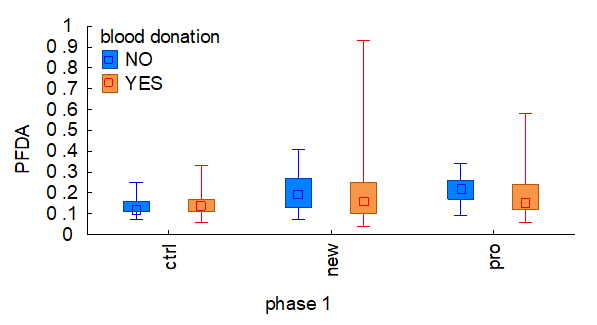 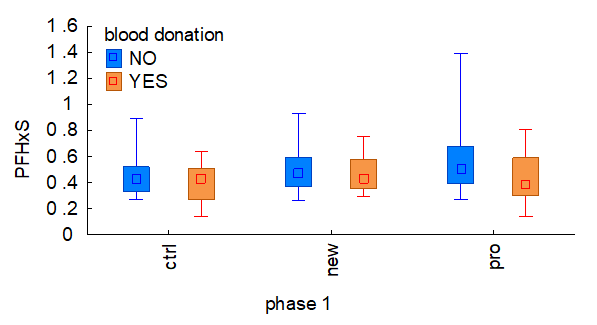 |
|  |
| 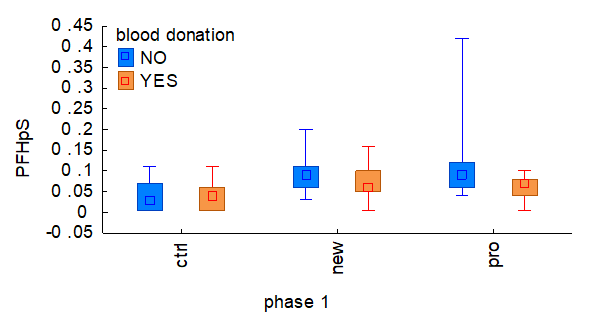 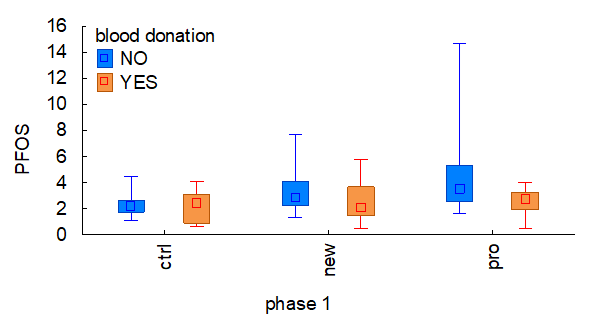 |
|  |

Figure SI18: Differences in PFAS concentrations for the blood donation parameter (YES/NO) (phase 1), median, 5-95^th^ (box), and 25-75^th^ (whiskers) percentiles. New = new trainees, prof = professionals, ctrl = control group.

## Cluster analysis

Cluster analyses were performed as an explanatory technique for determining cumulative PFAS and OH-PAH exposure. Ward's hierarchical clustering was used separately for OH-PAH and PFAS exposure to determine the similarity of participants with respect to the exposure factors (questionnaire variables). All non-binary responses from the questionnaires were first converted to binary data (1/0). This was done in such a way that the “never” frequency was converted to the value 0 and all other responses that indicated any frequency higher than “never” were converted to the value 1. A metric based on the Jaccard coefficient for binary data was used in the clustering. Then, serum PFAS levels and urine OH-PAH levels were compared among clusters using the Mann-Whitney U test.

All study participants (ctrl, new, and prof) from phase 1 (n = 165) were clustered according to selected factors that potentially affect the exposure to PFAS or OH-PAH. The variables with frequency information were transformed into binary variables (yes/no). Dental floss use, food supplements consumption, skiing, blood donation, diet (popcorn from microwave and fast food as a potential source of PFAS from food packages and fish consumption), use of GoreTex clothes/boots, use of firefighting foam, and contact with fire were used for the analysis of factors contributing to the PFAS exposure. Information about the former smoking, contact with fire, type of heating (coal or biomass vs gas or electricity), diet (consumption of grilled, fried, smoked, baked food or fast food), the population in the place of residence (cities vs suburban or villages) were used to assess the factors influencing OH-PAHs exposure.

In the cluster analysis of PFAS exposure factors, 5 clusters have been created (Figure SI19). For each cluster, the range and median concentration of the selected PFAS (with the highest sample detection) were calculated (Figure SI19). Cluster No. 1, having an increased level of some PFAS in comparison with other clusters, is characterized by an increased frequency of fire, foam, and GoreTex, and it is mostly composed of professional firefighters and new trainees (Figure SI19). Cluster No. 2 is represented mainly by professional firefighters and new trainees who did not use GoreTex clothes or firefighting foams. This cluster has similar median PFAS concentrations to clusters No. 4 and No. 5, which are mainly represented by the participants of the control group. However, the PFAS levels between clusters were not statistically significant, except for a statistically significant difference in PFNA levels found between clusters No. 1 and No.2.

| 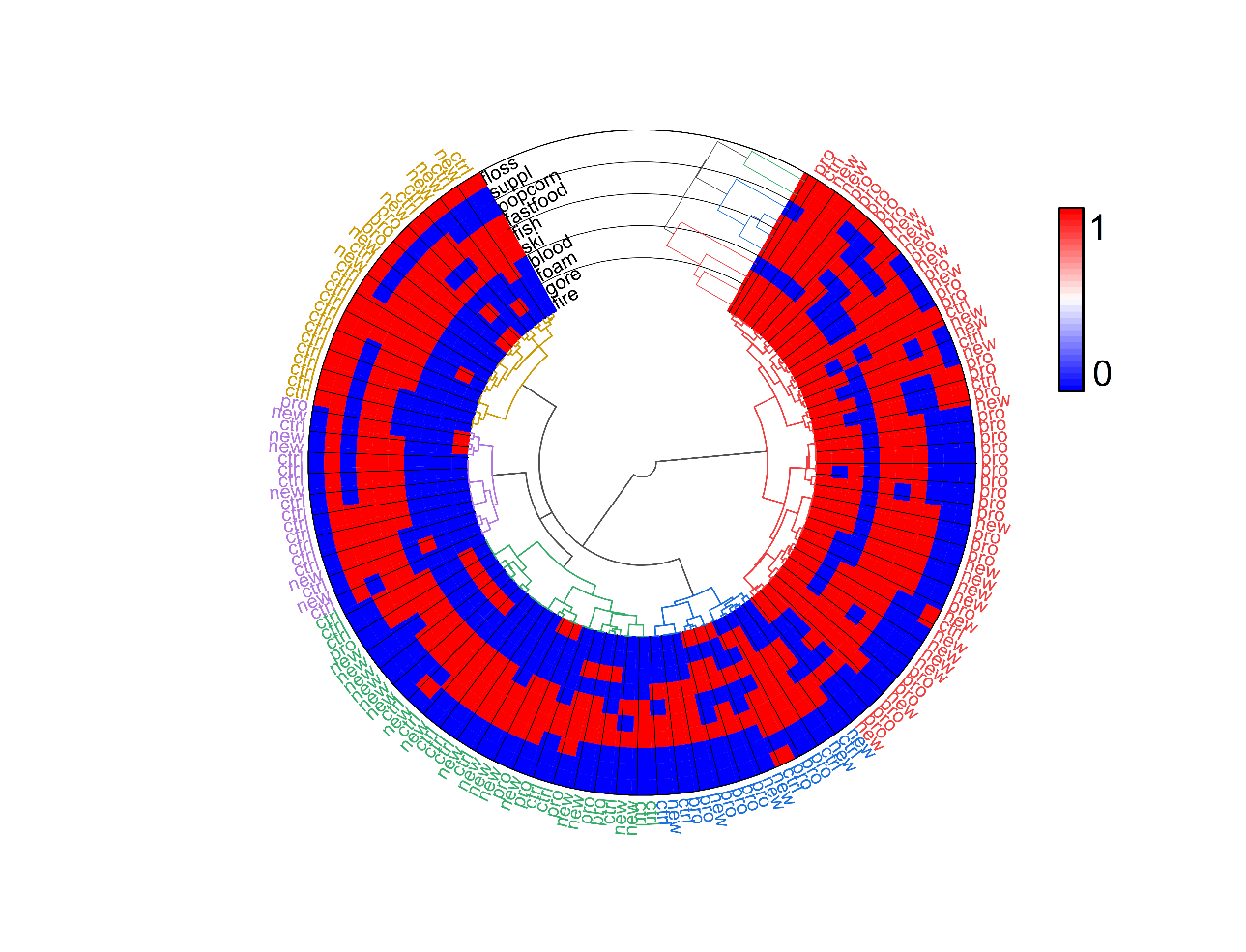 | |
| --- | --- |
| 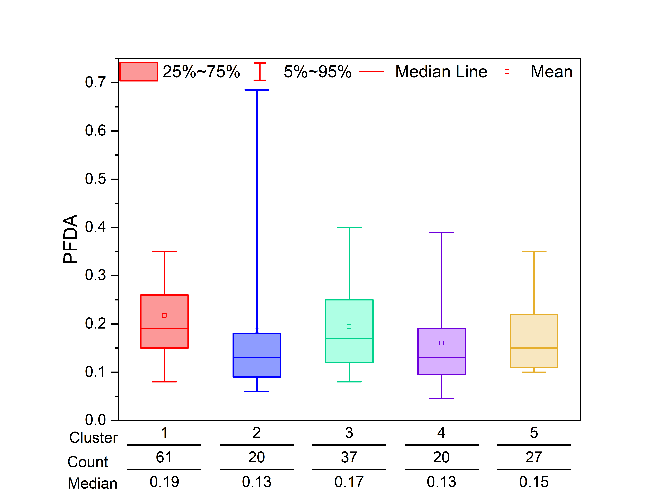 | 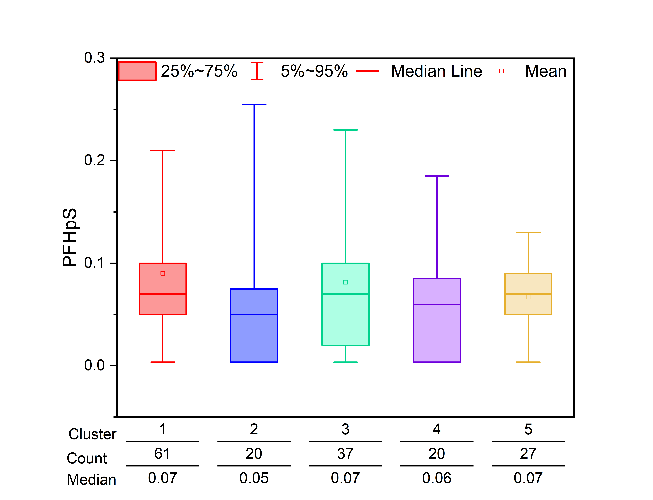 |
| 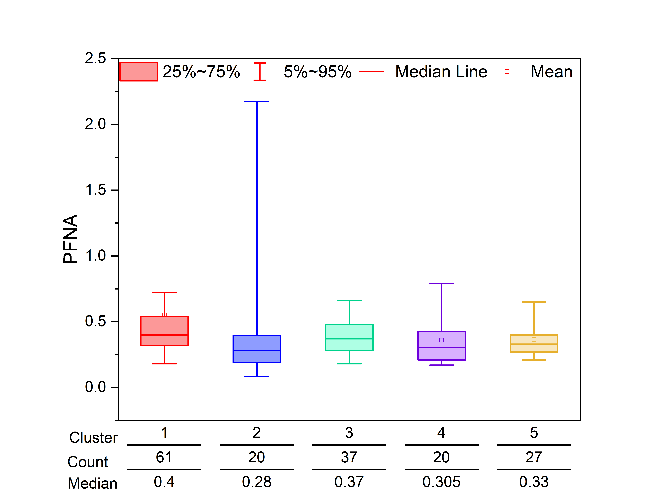 | 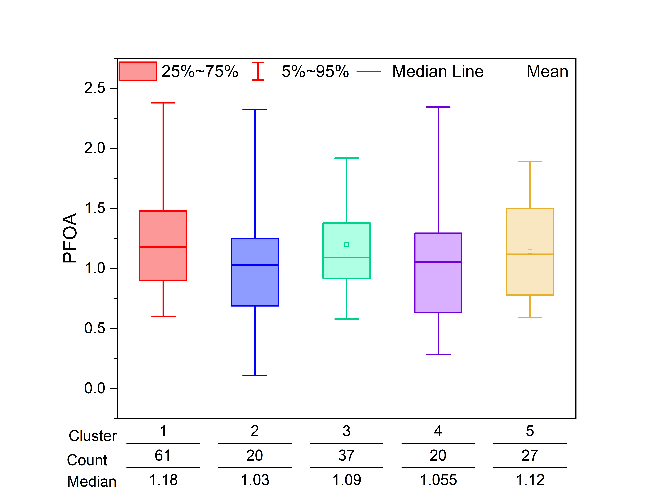 |
| 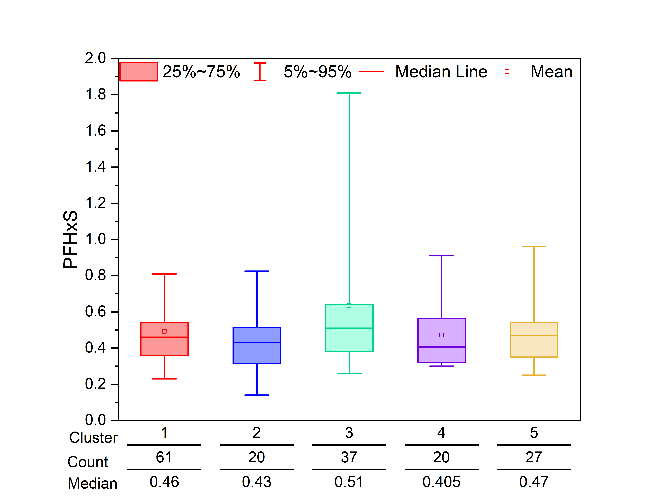 | 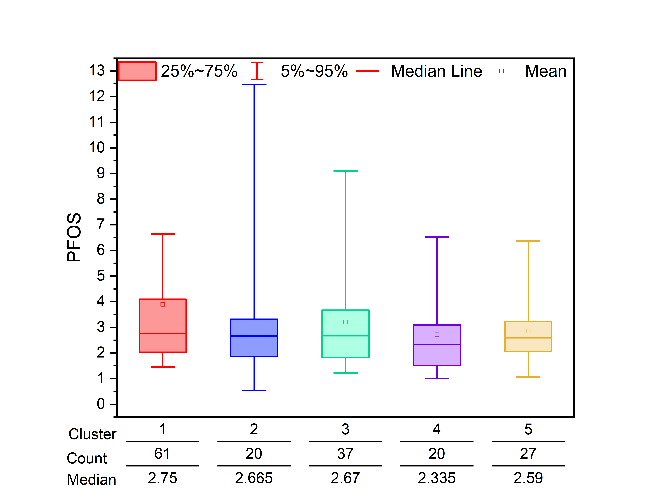 |

Figure SI19: Polar dendrogram of participants clustered according to the factors affecting PFAS levels (from questionnaires), in phase 1 and box and whiskers plot of PFAS concentrations in the clusters. No significant difference in PFAS concentrations was observed among clusters, except for the PFNA where cluster 2 levels were significantly different from other clusters.

When assessing the exposure factors potentially affecting the OH-PAH levels, participants were divided into 5 clusters (Figure SI20). There is no visible division according to firefighters and controls as in the case of PFAS and subcohorts are mixed in the clusters. There is little or no representation of exposure factors (except for the first cluster). No statistically significant difference was found for OH-PAH concentrations (individual or sum) between clusters.


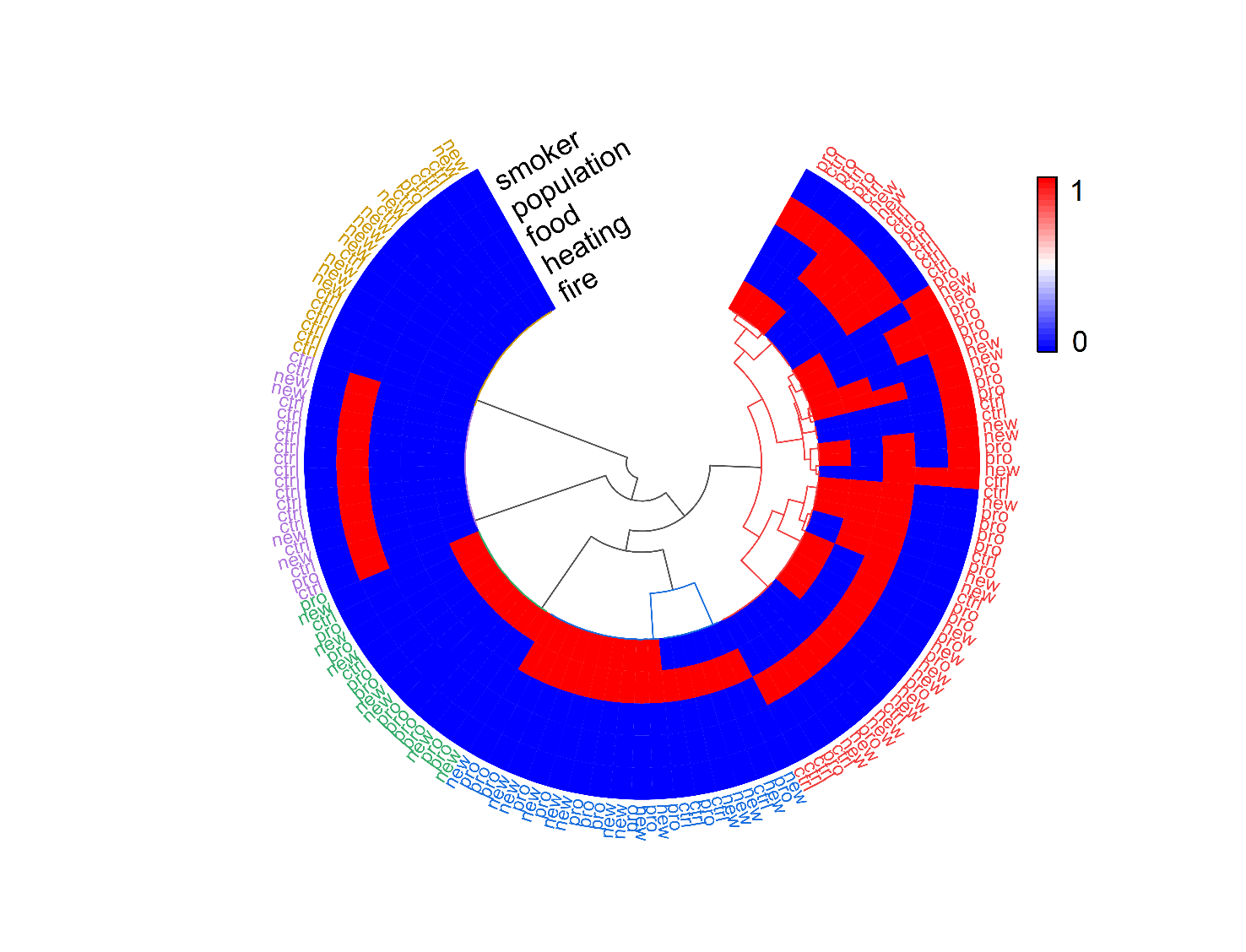


Figure SI20: Polar dendrogram of participants clustered according to the factors affecting OH-PAHs levels (from questionnaires), in phase 1 (before the new trainees were affected by the training).

## Literature

1. CDC’s National Center for Environmental Health (NCEH). Laboratory Procedure Manual. Analyte: Eight monohydroxy-polycyclic aromatic hydrocarbons: 1-hydroxynaphthalene, 2- hydroxynaphthalene, 2-hydroxyfluorene, 3- hydroxyfluorene, 1-hydroxyphenanthrene, 2- & 3-hydroxyphenanthrene, 1-hydroxypyrene. Matrix: Urine. 2013-2014

2. CDC’s National Center for Environmental Health (NCEH). Laboratory Procedure Manual. Analyte:Polyfluoroalkyl chemicals: Perfluorooctane sulfonamide, 2-(Nmethyl-perfluorooctane sulfonamido) acetate, 2-(N-ethylperfluorooctane sulfonamido) acetate, perfluorobutane sulfonate, perfluorohexane sulfonate, perfluorooc. Matrix: Serum. 2013

3. Dereziński P, Klupczyńska A, Sawicki W, Kokot ZJ. Creatinine determination in urine by liquid chromatography-electrospray ionization-tandem mass spectrometry method. *Acta Pol Pharm - Drug Res*. 2016;**73**(2):303–313.

4. Sauvé JF, Lévesque M, Huard M, et al. Creatinine and specific gravity normalization in biological monitoring of occupational exposures. *J Occup Environ Hyg*.;**12**(2):123–129.
